# Neutrophil Extracellular Traps (NETs) in health and disease

**DOI:** 10.1186/s43556-025-00337-9

**Published:** 2025-12-03

**Authors:** Asif Shahzad, Yueli Ni, Yinfeng Yang, Wenjing Liu, Zhuoran Teng, Honggang Bai, Xiangjie Liu, Yijian Sun, Jiaojiao Xia, Kun Cui, Qiuxin Duan, Zhe Xu, Jinshan Zhang, Zhe Yang, Qiao Zhang

**Affiliations:** 1 Department of Biochemistry and Molecular Biology, School of Basic Medical Sciences, Kunming Medical University, Kunming, Yunnan China; 2 Department of Clinical Laboratory, The Second Hospital of Jingzhou, Jingzhou, Hubei China; 3 School of Basic Medical Sciences, Qujing Medical College, Qujing, Yunnan China; 4 Departments of Pathology, The First Affiliated Hospital of Kunming Medical University, Kunming, Yunnan China

**Keywords:** Healthy states, Infectious disease, Autoimmune disease, Cardiovascular disease, Cancer

## Abstract

Neutrophil extracellular traps (NETs) are web-like structures composed of DNA, histones, and antimicrobial proteins that extend the defensive repertoire of neutrophils beyond classical phagocytosis and degranulation. Initially considered solely antimicrobial, NETs are now recognized as dynamic regulators of immunity, inflammation, and tissue remodeling. Their formation is orchestrated by the generation of reactive oxygen species, neutrophil elastase-mediated chromatin remodeling, and peptidyl arginine deiminase 4-driven histone citrullination. At the same time, clearance involves DNase activity and macrophage-mediated phagocytosis. In physiological contexts, NETs immobilize and kill pathogens, restrict biofilm formation, and coordinate immune cell crosstalk, thereby supporting host defense and repair. However, when NET formation or clearance becomes dysregulated, these structures drive a broad spectrum of pathologies. Aberrant NET activity has been implicated in infectious diseases (bacterial, viral, fungal), autoimmune disorders such as systemic lupus erythematosus, ANCA-associated vasculitis, rheumatoid arthritis, Gout, and psoriasis, cardiovascular disorders including atherosclerosis, thrombosis, acute coronary syndrome, Myocardial ischemia/reperfusion injury, hypertension, atrial fibrillation, heart failure, and viral myocarditis, as well as cancer progression, metastasis, and other inflammation-associated disorders such as asthma, Alzheimer’s disease, diabetes, and pregnancy-related complications. Advances in imaging, proteomics, and single-cell sequencing have expanded our ability to characterize NETs across contexts, revealing stimulus- and disease-specific heterogeneity. At the translational levels, therapies that inhibit NETs formation, promote their degradation, or regulate their release, including PAD4 and elastase inhibitors, DNase-based approaches, and antibody strategies, are under active investigation. By integrating these advances, this review provides a framework for translating NET biology into clinically relevant applications.

## Introduction

Neutrophils, the most abundant leukocytes in human circulation, constitute the first line of defense. Beyond their canonical functions of phagocytosis, degranulation, and production of reactive oxygen species, they deploy a distinctive antimicrobial mechanism: the release of neutrophil extracellular traps (NETs) [1, 2]. First described in 2004, these web-like structures of decondensed chromatin are composed of histones and antimicrobial proteins such as neutrophil elastase (NE) and myeloperoxidase (MPO), which act as extracellular scaffolds that immobilize and kill invading microbes. This discovery not only redefined neutrophil biology but also revealed a new dimension of host defense [3, 4].

It has become increasingly evident that NETs exhibit a dual nature, functioning as indispensable mediators of antimicrobial defense under physiological conditions, yet emerging as potent contributors to disease when their formation or clearance becomes dysregulated. Under controlled circumstances, NETs confine pathogens, limit microbial dissemination, and coordinate innate and adaptive immune responses. However, excessive or persistent NET release drives chronic inflammation and tissue damage by sustaining cytokine production and oxidative [5–7]. Uncontrolled NET accumulation or impaired degradation further promotes endothelial injury, vascular dysfunction, thrombosis, and autoimmunity, and has been increasingly implicated in tumor progression and metastasis [8]. Thus, NETs occupy a critical crossroads between protection and pathology, with their biological consequences determined by the balance between activation, regulation, and resolution.

Mechanistic advances have illuminated some of the molecular pathways driving NETs formation, including NADPH oxidase-dependent and -independent signaling, peptidyl arginine deiminase 4-mediated histone citrullination, and protease-driven chromatin remodeling [2, 9]. Yet major questions remain unresolved. How are NETs fine-tuned across diverse stimuli, from microbial signals to sterile inflammatory cues? By what routes are they effectively cleared in vivo? And how do they integrate with broader immune networks to balance antimicrobial defense with tissue homeostasis? Answering these questions is critical, as persistent ambiguities continue to complicate the interpretation of NETs biology across experimental and clinical settings [10, 11].

In this review, we provide recent progress in understanding NETs compositions, mechanisms of formation, and clearance pathways. We then explore their function in health and disease, with a particular emphasis on their pathogenic contributions across various conditions, including infectious, autoimmune, cardiovascular, and malignant diseases, as well as other clinically relevant settings such as asthma, Alzheimer's disease, hyperglycemia, and pregnancy-related complications. We also discuss advances in detection methods and therapeutic interventions. By integrating mechanistic insights with clinical observations, we aim to highlight how NETs biology can be translated into diagnostic and therapeutic opportunities, positioning NETs as both biomarkers and intervention points in a wide spectrum of human diseases. Unlike earlier reviews that primarily emphasized either the molecular underpinnings or clinical associations of NETs, we aim to provide a unified perspective that bridges these two dimensions. In doing so, this review seeks to clarify context-specific mechanisms, identify areas of translational opportunity, and outline how targeting NETs could reshape future therapeutic strategies.

## Biological characteristics of NETs

NETs represent a specialized antimicrobial defense mechanism characterized by unique molecular components, defined structural architecture, and tightly regulated formation and clearance processes. A detailed understanding of their composition, biogenesis, and resolution is crucial for elucidating how NETs contribute to both host protection and disease pathogenesis.

### Composition and structural features of NETs

NETs are web-like chromatin structures released by activated neutrophils, composed of decondensed DNA decorated with a broad spectrum of nuclear, granule-derived, and cytoplasmic proteins. First described as an antimicrobial mechanism that immobilizes and neutralizes pathogens [12]. NETs remain the most extensively characterized among extracellular traps, although related structures have been reported in eosinophils, mast cells, and macrophages [1]. The DNA backbone of NETs is interlaced with histones (H2A, H2B, H3, H4), which themselves possess antimicrobial activity, and coated with enzymes such as NE, MPO, proteinase 3 (PR3), together with cytoplasmic mediators such as calprotectin and lactoferrin [1]. Other proteins frequently associated with NETs include azurocidin (AZU1), cathelicidin LL-37, lysozyme C, and bactericidal/permeability-increasing protein B2 (BPIB2), reflecting the multifunctional nature of these structures [13]. The structural complexity of NETs reflects the coordinated release of nuclear and cytoplasmic constituents, including histones, NE, and MPO, which collectively mediate antimicrobial defense and immune modulation (Table 1). Beyond their protective roles, these components also participate in vascular inflammation, thrombosis, and autoimmunity, underscoring their dual biological impact.

**Table 1:** Molecular composition of NETs and their functional roles in health and disease

Components	Representative triggers	Primary source/ Localization	Associated diseases	Mechanism of action	Expected outcomes/functional implications	Refs
DNA (Chromatin backbone)	Oxidative burst signals, bacterial toxins, and calcium ionophores	Nuclear chromatin is released into the extracellular web-like fibers	Liver metastases	Chromatin decondensation, immune activation	Provides structural support for antimicrobial proteins; excessive release contributes to autoimmunity and tumor progression	[14]
			Systemic lupus erythematosus	Autoantibody formation, immune dysregulation		[15]
			*P. aeruginosa*	Bacterial entrapment, membrane disruption		[16]
Histone H2A (antimicrobial nuclear protein)	Cellular stress, microbial virulence factors	Chromatin-associated, released during NETosis	*Leishmania spp.*	Disrupts microbial membranes, enhances antimicrobial defense	Balance host protection with the risk of collateral tissue injury	[17]
			*E. coli and S. aureus*			[18]
Histones H2B (immune-modulatory histone)	ROS generation, bacterial stimulation	Nuclear chromatin fibers	*Leishmania spp.*	Modulates immune signaling pathways	Supports pathogen defense but may exacerbate inflammation in autoimmune conditions	[17]
Histones H4 (cytotoxic histone subtype)	Pro-inflammatory stressors, microbial toxins	Nuclear protein integrated into NETs fibers	*S. aureus* and Propionibacterium acnes	Antimicrobial, immune response	Protective antimicrobial effect, but contributes to cytokine storms and thrombosis in viral infections	[19]
			Influenza A virus	Inflammation, viral neutralization		[20]
			SARS-CoV-2	Immune response, viral neutralization		[21]
LL-37 (cationic antimicrobial peptide)	Microbial ligands, cytokine stimulation	Stored in granules, released into the extracellular space	*E. coli and S. aureus*	Membrane disruption, immunomodulation	Enhances microbial clearance, but excessive activity is linked to autoimmunity	[18]
			*C. albicans*			[22]
			Systemic lupus erythematosus			[23]
			Influenza A virus			[24]
Neutrophil elastase (NE, granule-derived protease)	Pro-inflammatory mediators, bacterial toxins	Azurophilic granules, nucleus, and extracellular space	*Shigella* and *Yersinia*	Protease-mediated bacterial degradation	Contributes to host defense, but also tissue damage, and cancer progression	[25]
			*C. albicans*	Fungal cell wall degradation		[22]
			Lung adenocarcinomas	Tissue remodeling, inflammation		[26]
			ANCA-associated vasculitis	Viral clearance, immune response		[27]
			respiratory syncytial virus	Airway inflammation, immune response		[28]
Myeloperoxidase (MPO, oxidative enzyme)	ROS burst, microbial triggers, and inflammatory cytokines	Azurophilic granules, NETs structures	Bacterial infection	Reactive oxygen species production	Essential for antimicrobial action; excessive activity causes oxidative stress and tissue injury	[29]
			*C. albicans*	Antifungal reactive oxygen species		[30]
			ANCA-associated vasculitis	Viral clearance, oxidative stress		[31]
			HIV-1			[32]
Azurocidin (cationic antimicrobial mediator)	Microbial recognition, pro-inflammatory signaling	Cytoplasmic granules are released extracellularly	Host defense	Enhances antimicrobial activity, modulates immune responses	Supports clearance of microbes, may promote inflammation if dysregulated	[29]
Cathepsin G (serine protease)	Bacterial stimulation, ROS pathways	Granules, nucleus, extracellular compartment	Host defense	Proteolytic activity, immune modulation	Pathogen clearance, but excessive proteolysis damages host tissues	[33]
			respiratory syncytial virus	Inflammation, viral clearance		[28]
Proteinase 3 (PR3, serine protease)	Pro-inflammatory signaling, microbial ligands	Granules, cell surface, extracellular space	Host defense	Protease activity, antimicrobial activity, and inflammatory signaling	Protective against infection, but contributes to vasculitis and inflammation	[33]
			ANCA-associated vasculitis			[31]
			respiratory syncytial virus			[28]
Neutrophil defensing (α-defensins)	Pathogen-associated signals	Released from cytoplasmic granules into the extracellular space	*S. aureus*	Antibacterial, immune response	Clears microbes efficiently, but sustained release drives autoimmunity	[34]
			Systemic lupus erythematosus	Inflammation, immune regulation		[23]
			Influenza A virus	Antiviral response, immune activation		[24]
			HIV-1			[32]
Heat shock protein 72	Cellular stress, infection, inflammation	Cytoplasm, nucleus, and extracellular	*M. tuberculosis*	Chaperone activity, immune modulation	Enhances stress response and pathogen clearance, may promote autoimmunity	[35]
Interstitial collagenase (MMP1)	Infection-driven inflammation	Extracellular compartment	Fungi infections	Tissue remodeling, immune response	Facilitates immune cell migration, but excessive activity drives tissue injury	[36]
Lactrotransferrin (iron-binding glycoprotein)	Microbial infection, oxidative signals	Cytoplasmic granules, extracellular space	*Candida* species	Sequesters iron, inhibits microbial growth	Prevents microbial proliferation, and iron restriction may also stress host cells	[37]
Calprotectin (S100A8 /S100A9 complex)	Infection-induced activation	Cytoplasm, membrane, and extracellular traps	*C. albicans*	Chelates ZN2 + /Mn2 + antifungal activity	Restricts fungal growth, but elevated systemic levels drive inflammation	[38]
			*A.fumigatus*			[39]
Matrix metalloproteinase 9 (MMP9)	LPS stimulation, tumor-associated inflammation	Extracellular compartment	Cancer progression, metastasis	ECM degradation, tissue remodeling	Promotes metastasis and angiogenesis	[40]
α-enolase (glycolytic enzyme/autoantigen)	Cellular stress, inflammatory triggers	Cytoplasm, extracellular NETs	Systemic lupus erythematosus	Autoantigen, pro-inflammatory activity	Amplifies autoantibody generation, chronic inflammation	[41]
Annexin A1 (anti-inflammatory mediator)	Stress signals, microbial components	Cytoplasm, NETs structures	Systemic lupus erythematosus	Immune modulation, anti-inflammatory activity	Suppresses excessive inflammation; dysregulation linked to autoimmunity	[41]
High-mobility group box 1 (HMGB1; nuclear DNA-binding protein)	Cellular stress, necrosis, and infection	Nucleus, cytoplasm, NETs fibers	Systemic lupus erythematosus	Pro-inflammatory mediator, DAMP signaling	Amplifies immune activation, contributes to systemic inflammation	[42]
Lysosomal membrane protein-2 (LAMP2)	Viral infection, oxidative stress	Lysosomal membranes, extracellular vesicles	ANCA-associated vasculitis	Facilitates viral entry, immune signaling	Enhances viral pathogenesis but may also prime immune defense	[43]

*Abbreviations: AVV* ANCA-associated vasculitis, *C. albicans Candida albicans*, *E. coli Escherichia coli*, *IAV* influenza A virus, *LPS* lipopolysaccharide, *MMP9* matrix metalloproteinase 9, *NE* neutrophil elastase, *PMA* phorbol 12-myristate 13-acetate, *RSV* respiratory syncytial virus, *S. aureus Staphylococcus aureus*, *SLE* systemic lupus erythematosus

Advances in proteomics have expanded our understanding of NETs composition, revealing that their molecular repertoire is not fixed but shaped by the inducing stimulus and disease context. Early studies of PMA-induced NETs identified 24 core proteins [44], whereas recent high-throughput mass spectrometry has identified over 330 proteins, of which 74 are consistently detected across different stimuli and models [45]. Stimulus-specific differences are increasingly recognized: For example, dynamin (DNAH5), HSPA1B, and RPS27 are present in both PMA- and the calcium ionophore-induced NETs, while lysosome-associated membrane protein 2 (LAMP2) is preferentially enriched in lipopolysaccharide (LPS)-induced NETs [3]. Pathogen-driven variation has also been documented; NETs triggered by different *Pseudomonas aeruginosa* strains share 33 conserved proteins but differ by up to 50 additional components, illustrating pathogen-specific modulation [46]. In clinical settings, NETs derived from patients with autoimmune diseases such as systemic lupus erythematosus (SLE), ANCA-associated vasculitis (AAV), and rheumatoid arthritis (RA) appear broadly similar under standardized stimulation [47]. However, more extensive comparisons reveal clear distinctions between NETs associated with infectious versus sterile inflammatory states [48]. Collectively, this proteomic diversity underscores that NETs composition is determined primarily by the nature of the stimulus rather than by the intrinsic characteristics of neutrophils, and highlights the versatility of NETs in mediating both host defense and pathological processes across infectious, autoimmune, thromboinflammatory, cardiovascular, malignant, and neurodegenerative diseases [49, 50].

### Mechanisms of NETs formation

NETs formation, or NETosis, is initiated by a diverse range of stimuli that broadly encompass microbial components, pharmacological activators, and host-derived inflammatory or tumor-associated signals. While the initiating triggers vary, they converge onto conserved molecular pathways that orchestrate chromatin decondensation, nuclear membrane breakdown, and extracellular release of DNA–protein complexes [12, 51]. Importantly, two overarching modes of NETosis have been identified: ROS-dependent and ROS-independent, with stimulus-specific variations reflecting the context of infection, inflammation, or malignancy.

Microbial stimuli such as Lipopolysaccharide (LPS), a major Gram-negative bacterial component, exemplify how pathogen-associated molecules drive NETs release [52]. LPS acts indirectly through platelet TLR4, which engages IRF1-dependent signaling and platelet-neutrophil crosstalk involving ERK, PI3K, and Src kinases. This pathway differs from the PKC-ROS axis triggered by pharmacological activators such as phorbol 12-myristate 13-acetate (PMA) [53, 54]. PMA represents the most robust experimental inducer of NETosis, activating PKC and the RAF-MEK-ERK cascade, thereby stimulating NADPH oxidase-mediated ROS generation [12, 54]. Its effects are abrogated by pharmacological blockade of PKC or NADPH oxidase, underscoring the centrality of ROS in this pathway [55, 56].

In parallel, host-derived inflammatory and tumor-associated mediators provide another layer of regulation. High Mobility Group Box 1 (HMGB1), released by necrotic and activated immune cells, triggers NETosis via TLR4, RAGE, and MAPK signaling, and in cancer settings, also engages RIPK1-TNF signaling to couple NETs with tumor progression [54, 57, 58]. Cathepsin C (CTSC), a cysteine protease secreted by tumors, promotes NETs release through activation of neutrophil serine proteases such as PR3 and NE, linking it to 1β-p38 MAPK signaling [9, 54, 59]. Similarly, Granulocyte Colony-Stimulating Factor (G-CSF) primes neutrophils for heightened NETosis in inflammatory and malignant states, although the precise downstream mediators remain incompletely defined [55, 59–61]. Among chemokines, Interleukin-8 (IL-8) is especially potent; by binding to CXCR1/2 [62]. IL-8 activates Src-PI3K-Akt-ERK and p38 MAPK cascades, driving NETs formation in cancers, including non-small cell lung cancer (NSCLC), melanoma, and gastric cancer. Importantly, CXCR1/2 antagonists such as reparixin effectively suppress IL-8-driven NETosis, highlighting translational potential [54, 63–65].

**Fig. 1:**
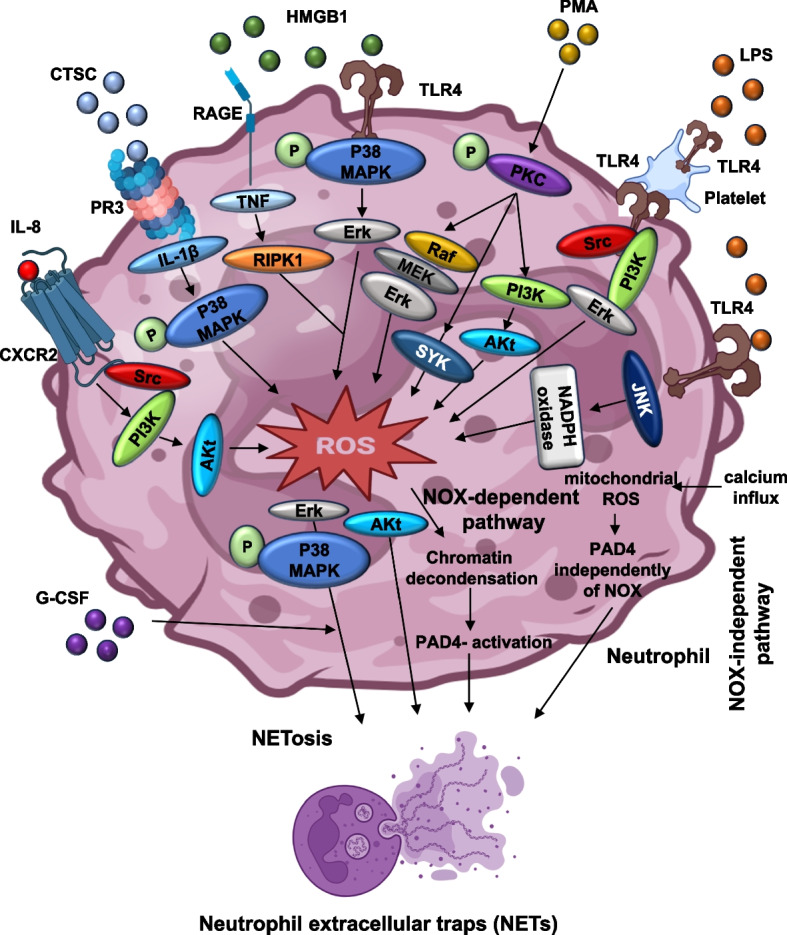
Schematic representation of NETosis signaling pathways induced by various stimuli. This figure illustrates the key molecular pathways involved in the induction of neutrophil extracellular traps (NETs). The diagram highlights various NET-inducing stimuli, including LPS, PMA, HMGB1, G-CSF, IL-8, and CTSC, that initiate signaling via pattern recognition receptors, such as toll-like receptors (TLRs). These activate PKC, leading to downstream events that differ between NADPH oxidase (NOX)-dependent and NOX-independent NETosis. In the NOX-dependent pathway, ROS generated by NOX facilitates nuclear delobulation (i.e., loss of the normal multi-lobed nuclear structure) and NE/MPO translocation to the nucleus. PAD4 is activated to citrullinate histones, enabling chromatin decondensation. In the NOX-independent pathway, calcium influx and mitochondrial ROS activate PAD4 independently of NOX. These parallel routes converge on nuclear envelope breakdown and NETs release. Abbreviations: NETs: neutrophil extracellular traps, LPS: lipopolysaccharide, PMA: phorbol 12-myristate 13-acetate, HMGB1: High mobility group box 1, G-CSF: granulocyte colony-stimulating factor, IL-8: interleukin-8, CTSC: cathepsin C, ROS: reactive oxygen species, PKC: protein kinase C, NE: neutrophil elastase, PAD4: peptidyl arginine deiminase 4. Note: Figure created using Microsoft PowerPoint and Adobe Illustrator AI. Visual elements were adapted from publicly available scientific graphics and BioRender under fair academic use. No commercial license was obtained

At the molecular level, NETosis unfolds through highly orchestrated intracellular events. In the ROS-dependent pathway, activation of PKC, Syk, and small GTPases leads to NADPH oxidase-driven ROS production. ROS promote the translocation of NE and MPO from azurophilic granules into the nucleus, where NE degrades histones and MPO facilitates chromatin relaxation [12, 54]. Concurrently, PAD4 catalyzes histone citrullination, further loosening chromatin and driving nuclear swelling. Eventually, rupture of nuclear and plasma membranes releases decondensed chromatin decorated with histones, NE, MPO, and antimicrobial proteins [66, 67]. In contrast, ROS-independent pathways, triggered by calcium ionophores or receptor-mediated signaling, rely on calcium influx and PAD4 activation without NADPH oxidase involvement [10, 11, 68]. Recent work has revealed that specific stimuli such as HMGB1 and CTSC initiate distinct signaling modules, underscoring the context-dependent heterogeneity of NETosis across infection, autoimmunity, thrombosis, and cancer [69, 70]. This mechanistic framework highlights that while NETosis is unified by core processes of chromatin decondensation and nuclear rupture, the diversity of upstream signals, microbial, pharmacological, or host-derived, confers stimulus-specific signatures. This complexity offers both challenges and opportunities for therapeutic targeting, as selective modulation of pathogenic NETosis may be achievable without compromising essential antimicrobial defense. Figure 1 schematically summarizes these converging pathways and their points of divergence.

### Clearance mechanism of NETs

Neutrophils, the first responders of the innate immunity, are rapidly mobilized to sites of infection or sterile injury, where they neutralize pathogens and modulate the local inflammatory milieu. Their antimicrobial arsenal includes phagocytosis, degranulation, production of reactive oxygen species (ROS), and the release of NETs [71, 72]. After fulfilling their effector functions, most neutrophils undergo apoptosis and are subsequently cleared by macrophages or tissue-resident phagocytes, while a subset may return to the bone marrow through reverse transmigration. This tightly regulated turnover preserves tissue homeostasis and prevents excessive collateral injury.

Beyond their microbial function, neutrophils also orchestrate adaptive immune responses by releasing cytokines such as B-cell activating factor (BAFF) and a proliferation-inducing ligand (APRIL), which stimulate B-cell and dendritic cell activity. NETs reinforce this cross-talk by functioning as immunostimulatory scaffolds. However, if not efficiently dismantled, NETs persist within tissues, where they serve as potent amplifiers of inflammation and immune activation [72].

Effective resolution of inflammation depends on the timely clearance of NETs, a process largely mediated by deoxyribonuclease 1 (DNase 1), which digests chromatin into smaller fragments suitable for phagocytic uptake by macrophages, particularly those of the M2 subtype [9]. Additional regulatory layers include the antimicrobial peptide LL-37, which enhances DNA degradation, and DNase 1-like 3 (DNase 1L3), which is especially critical in immune complex-rich microenvironments. Disruption of these pathways through either excessive NETs release or impaired clearance promotes chronic inflammation and contributes to the development of autoimmunity [9, 73].

Defective NETs clearance is particularly pathogenic in SLE. Mechanistically, DNase 1 deficiency, neutralizing anti-DNase1L3 antibodies, and DNASE1L3 mutations converge to impair extracellular chromatin degradation, facilitating NETs accumulation, immune complex formation, and lupus nephritis [74]. In parallel, Low-density granulocytes (LDGs), a neutrophil subset enriched in SLE, exhibit a strong propensity for NETs release and heightened proinflammatory cytokine production, exacerbating tissue injury. Persistent NETs also activate the complement cascade, driving C3a/C5a generation and leukocyte recruitment, which sustain vascular injury, tissue necrosis, and systemic autoimmunity [47].

## Function of NETs in healthy states

Beyond their antimicrobial activity, NETs contribute to diverse physiological processes essential for host defense and tissue homeostasis. They restrict pathogen dissemination, modulate immune signaling by shaping macrophage and T cell responses, and support biofilm control. Under tightly regulated conditions, NETs also aid tissue repair and remodeling, illustrating their dual role as both antimicrobial effectors and immunomodulatory scaffolds in maintaining health.

### Host defense against pathogen invasion

**Fig. 2:**
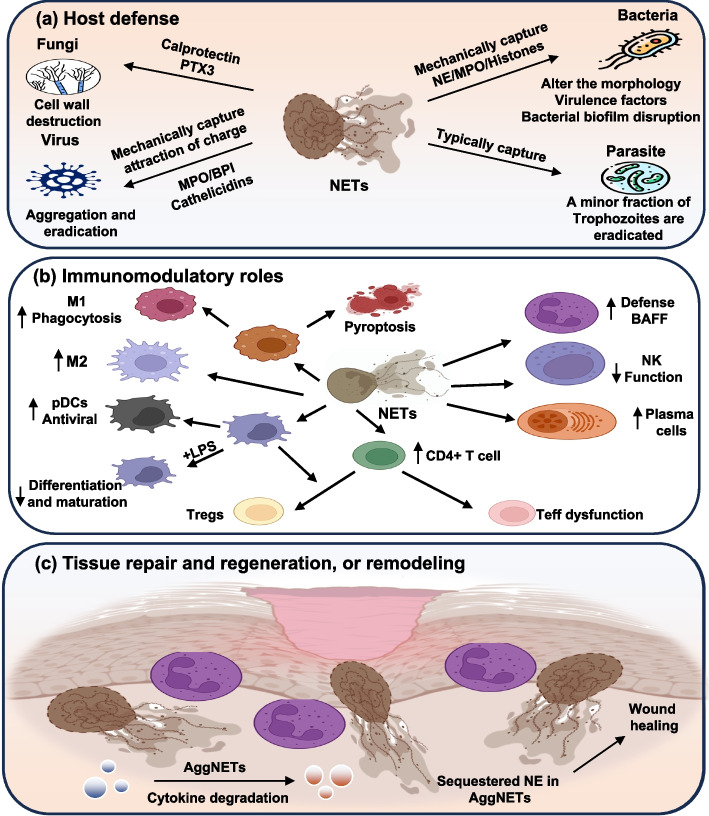
Neutrophil extracellular traps (NETs) in health. NETs contribute to host defense and tissue homeostasis through multiple mechanisms: **a** Host defense: NETs capture and immobilize invading pathogens. Proteins embedded with the DNA-histone scaffold, such as NE, MPO, and histones, disrupt microbial membranes, neutralize virulence factors, and facilitate pathogen killing. **b** Immunomodulatory roles: NETs enhance neutrophil antimicrobial defense and shape adaptive immunity. They promote macrophage polarization toward pro- or anti-inflammatory phenotypes, induce pyroptosis in infected cells, and facilitate pDC differentiations to boost antiviral responses. **c** Tissue repair and regeneration, or remodeling: aggregated NETs contribute to inflammation resolution and wound healing by degrading pro-inflammatory cytokines and sequestering NE, thereby preventing excessive extracellular matrix proteolysis and tissue damage. Abbreviations: NETs: neutrophil extracellular traps; NE: neutrophil elastase; MPO: myeloperoxidase; pDC: plasmacytoid dendritic cell; NK: natural killer; aggNETs: aggregated neutrophil extracellular traps. Note: Figure created using Microsoft PowerPoint and Adobe Illustrator AI. Visual elements were adapted from publicly available scientific graphics and BioRender under fair academic use. No commercial license was obtained

NETs are a crucial component of innate immune defense, complementing classical antimicrobial mechanisms such as phagocytosis and degranulation. In response to bacterial, viral, fungal, and parasitic infections, neutrophils release web-like structures that entrap pathogens, concentrate antimicrobial proteins, and generate localized microbicidal environments that restrict dissemination and promote clearance (as shown in Fig. 2a) [75, 76]. The DNA-histone backbone, decorated with proteolytic enzymes and antimicrobial peptides, not only immobilizes invading organisms but also facilitates their degradation.

Importantly, NETs also act in the context of biofilm-associated infections, where they disrupt microbial architecture through NE and MPO [77]. Both NADPH oxidase (NOX)-dependent and PAD4-driven NOX-independent pathways contribute to this defense. Yet, the interaction between NETs and biofilms illustrates their functional duality: while they can dismantle biofilm structure, NETs also provide extracellular DNA that serves as a scaffold for microbial adhesion and horizontal gene transfer, thereby inadvertently sustaining persistence [78, 79]. Certain pathogens further evade NETs killing by secreting nucleases that degrade the DNA backbone [80, 81]. These observations underscore the complexity of NET-mediated host defense, balancing protective antimicrobial functions with potential unintended support of chronic infection.

### Immunomodulatory roles of NETs

Beyond their direct antimicrobial effects, NETs exert a profound influence on the regulation of innate and adaptive immunity. NETs interact with macrophages to modulate inflammasome activity, where engagement of the NLRP3 pathway leads to IL-1β and IL-18 release, reinforcing a proinflammatory cycle that feeds back into additional NETs production (as shown in Fig. 2b) [56]. At the adaptive interface, NETs support T helper 17 (Th17) differentiation through TLR2-STAT3 signaling, enhancing IL-6 and IL-17 release, thereby amplifying neutrophil-driven inflammation [82]. In parallel, NETs can act as immunomodulatory scaffolds that sequester or enzymatically process cytokines: they preserve IL-1α activity, degrade IL-1β, and regulate IL-33 and IL-36 signaling, creating context-dependent outcomes [83]. Collectively, these mechanisms highlight the dual role of NETs, facilitating immune resolution and tissue repair when tightly regulated, yet perpetuating chronic inflammation when their clearance fails [33, 83].

### Tissue repair and regeneration, and remodeling

Increasing evidence supports a role for NETs in wound healing and tissue remodeling [26]. Under physiological conditions, controlled NETs release contributes to the resolution of inflammation and supports the transition from injury to repair [36, 84]. Through sequestration of proteases and modulation of local cytokine activity, NETs help balance pro- and anti-inflammatory signals during the early phases of healing [29]. Persistent accumulation of NETs leads to excessive ROS generation and release of cytotoxic granule proteins, which exacerbate tissue injury, delay wound closure, and contribute to fibrosis (as shown in Fig. 2c) [40]. Experimental targeting of NETs components has been shown to accelerate repair in models of chronic wounds, suggesting that precise modulation of NETs activity may represent a promising therapeutic approach to enhance regeneration while minimizing tissue injury.

## Roles of NETs in diseases

Neutrophil extracellular traps are increasingly recognized as central players in the pathogenesis of a wide spectrum of diseases, extending well beyond their classical antimicrobial roles. In infectious diseases, NETs form a critical first line of defense against bacterial, fungal, viral, and parasitic pathogens, yet excessive or evasion-prone NETs responses can promote chronic infection and tissue damage [80]. In autoimmune disorders such as SLE, ANCA-associated vasculitis, RA, Gout, and psoriasis, defective NETs clearance and exposure of autoantigens like citrullinated histones, MPO, and PR3 contribute directly to loss of immune tolerance and sustained inflammation [63]. Cardiovascular diseases, including atherosclerosis and thrombosis, are strongly linked to NET-driven immunothrombosis, where chromatin fibers decorated with proteases and histones act as scaffolds for platelet adhesion and coagulation factor activation [85]. In cancer, NETs play multifaceted roles, including primary tumor progression, metastatic dissemination, organotropism, and therapy resistance, often through interactions with tumor-secreted factors and immunosuppressive signaling pathways [4, 86]. Emerging evidence implicates NETs in a diverse array of conditions ranging from asthma and chronic obstructive pulmonary disease to neuroinflammatory disorders such as Alzheimer's disease, metabolic dysregulation in diabetes and hyperglycemia, and pregnancy-associated complications, underscoring their broad clinical relevance [47, 87]. These findings establish NETs as both protective and pathogenic effectors, whose context-dependent roles vary across disease categories, highlighting them as attractive yet challenging therapeutic targets.

### Infectious diseases

NETs play a crucial role in host defense against bacterial, fungal, and viral infections by trapping and neutralizing pathogens. In the oral cavity, NETs contribute to antimicrobial defense by preventing bacterial adhesion and invasion, particularly in periodontal diseases. Studies showed that at least 19 oral bacteria associated with periodontitis can induce NETs formation, highlighting their role in maintaining oral mucosal homeostasis [28]. NETs interact with salivary components to enhance antimicrobial properties while also acting as a structural scaffold for tissue repair, ensuring a protected wound-healing environment in the oral mucosa.

#### NETs in bacterial infections

NETs represent a vital component of innate immune defense, functioning to physically entrap and neutralize bacterial pathogens. These structures consist of decondensed chromatin frameworks interlaced with antimicrobial proteins such as histones (e.g., H2A), NE, MPO, and serine proteases. By immobilizing bacteria and disrupting their virulence, these molecules can damage bacterial membranes or degrade critical virulence determinants, thereby contributing to host protection [88, 89].

Bacterial pathogens can both trigger NETs formation and employ diverse strategies to evade or degrade these structures. Pathogenic *Escherichia coli* strains, unlike their nonpathogenic counterparts, induce strong NETosis, suggesting that virulence-associated molecular patterns are essential for activating neutrophils [89]. In *Shigella flexneri*, NETs containing NE degrade key virulence proteins such as IcsA and IpaB, leading to bacterial inactivation [90, 91]. *Streptococcus pneumoniae* and *S. pyogenes* not only stimulate NETs release but also evade entrapment via the production of DNases (e.g., EndA, Sda1) that dismantle the chromatin scaffold or by expressing capsular polysaccharides that limit NETs binding. Additionally, the group A *streptococcal* M1 protein promotes NETs formation while simultaneously binding LL-37, reducing its bactericidal potency. *Staphylococcus aureus* triggers rapid NETosis but produces catalase and nucleases to neutralize ROS and degrade NETs structures, respectively, a mechanism particularly efficient in *methicillin-resistant S. aureus (MRSA*). *Mycobacterium tuberculosis*, including both virulent (H37Rv) and less virulent strains, induces ROS generation and NETs release in vitro [89, 92]. However, while NETs can trap the pathogen, bacterial killing is minimal, indicating a containment rather than a clearance role. *Klebsiella pneumoniae* exhibits poor NETs induction in isolated conditions but robustly stimulates NETs formation during lung infections [93, 94]. Experimental models demonstrate that neutrophils deficient in NE or MRP14 form fewer NETs and fail to limit bacterial dissemination, whereas A2B receptor-deficient mice show enhanced NETs production and improved pathogen clearance [89, 95, 96].

Despite the absence of highly specific NETs biomarkers for bacterial infections, several circulating molecules show promise for clinical application. Circulating free DNA (cfDNA) and MPO-DNA complexes are frequently elevated in septic patients, correlating with disease severity and mortality risk [97, 98]. Preclinical studies indicate that recombinant DNase therapy can mitigate NET-mediated tissue damage. Presepsin (sCD14-ST), though not NET-specific, serves as an indicator of immune activation during bacterial infections and may reflect NET-driven inflammatory responses. Additionally, NET-associated proteins such as calprotectin (S100A8/A9) and lipocalin-2 (NGAL) are released in significant quantities during bacterial infection, contributing to pathogen sequestration and inflammatory signaling, and may act as systemic biomarkers. NET-dependent antimicrobial action against bacterial pathogens is multifactorial, combining physical entrapment with the enzymatic degradation of virulence factors [99–101].

#### NETs in fungal infections

Neutrophils are central players in the innate immune defense against fungal pathogens, and NETs represent a key component of their antifungal armamentarium. Despite their significance, only a limited range of fungal species has been systematically evaluated for their ability to induce NETs formation and for susceptibility to NET-mediated killing [102, 103]. Among these, *Candida albicans* is the most prevalent fungal commensal, asymptomatically colonizing the skin and mucosal surfaces in approximately 30–50% of healthy individuals. Under immunocompromised conditions, *Candida spp.* can become highly opportunistic, causing systemic infections with mortality rates reaching up to 40% [103, 104]. The virulence of *C. albicans* is closely linked to its dimorphic nature, where the yeast form facilitates systemic dissemination, while the hyphal form is essential for persistence and tissue invasion. As hyphae are too large for phagocytic uptake, NETs release offers an effective extracellular killing mechanism. Multiple studies have confirmed that NETs are capable of killing both yeast and hyphal morphotypes of *C. albicans * [105, 106]*.*

Molecular dissection of NETs' antifungal activity has identified calprotectin as a major NET-associated biomarker and antifungal effector. Calprotectin, a cytoplasmic protein complex composed of S100A8 and S100A9 subunits, is released during NETosis both in soluble form and bound to chromatin fibers [107]. Its antifungal function largely derives from chelation of essential micronutrients such as Mn^2+^ and Zn^2+^, thereby restricting fungal growth. The pivotal role of calprotectin in antifungal immunity is underscored by the heightened susceptibility of calprotectin-deficient mice to both subcutaneous and pulmonary candidiasis [108]. In *Aspergillus nidulans* infections, calprotectin-deficient mice exhibit increased vulnerability, with protection shown to depend on the presence of both S100A8 and S100A9 subunits. Interestingly, at low concentrations, calprotectin exerts fungistatic effects, whereas at high concentrations it induces nutrient starvation, leading to fungal death [109]. The clinical relevance of NET-mediated antifungal defense is evident in chronic granulomatous disease (CGD) patients, who have defective NADPH oxidase activity, resulting in impaired ROS production and compromised NETs release. These patients often experience recurrent *Aspergillus* infections. Gene therapy that restores NADPH oxidase function has been shown to reinstate NETs formation and calprotectin release, enabling effective clearance of *A. nidulans* infections [103, 110].

The upstream recognition events governing NETosis in response to fungi are still incompletely understood. In *A. fumigatus*, NETs induction occurs independently of fungal viability, as both inactivated conidia and hyphae can trigger NETs release. The efficiency of NETs induction appears to be influenced by fungal surface structures [103, 111]. For example, conidia express the hydrophobin protein RodA, which masks immunostimulatory fungal components and reduces NETs formation compared to hyphae. RodA-deficient conidia display significantly greater NET-inducing capacity, suggesting that RodA inhibits exposure of key NET-triggering ligands. Nevertheless, *A. fumigatus* conidia are primarily eliminated via phagocytosis rather than NETosis, while *A. nidulans* conidia are susceptible to NET-mediated killing, albeit less efficiently than hyphae [111]. Beyond calprotectin, NETs are enriched with other antimicrobial proteins, including NE, MPO, and lactoferrin, which collectively disrupt fungal membranes and sequester essential nutrients, further impairing fungal growth. Mutations in JAGN1, which lead to reduced MPO expression within NETs, impair antifungal killing capacity, highlighting MPO as another critical NET-associated biomarker for fungal clearance [55, 112, 113]. Conversely, certain fungi, such as *Pneumocystis jirovecii*, appear capable of evading NET-mediated immunity by interacting with NETs components in ways that suppress host responses. Understanding such immune evasion strategies holds promise for the development of next-generation antifungal therapies that enhance NET-mediated killing while preventing pathogen escape [114, 115].

#### NETs in viral infections

Traditionally, neutrophils have not been considered primary effector cells in antiviral immunity, and relatively few studies have explored their role in viral defense. However, emerging evidence indicates that NETosis can be induced during viral infections and may contribute significantly to host protection [48, 89]. For example, human immunodeficiency virus type 1 (HIV-1) has been shown to trigger NETosis via a cell death-dependent pathway, although the requirement for ROS, MPO, and NE in this process remains unresolved. NETs can physically capture and neutralize negatively charged HIV virions, thereby reducing viral infectivity. Despite this, HIV has evolved mechanisms to suppress NETs formation; it manipulates neutrophil activation indirectly by engaging the dendritic cell-specific ICAM-3 grabbing non-integrin (DC-SIGN, CD209) receptor on dendritic cells via its envelope glycoprotein gp120 [116, 117]. This interaction stimulates interleukin-10 production, which inhibits NETs release. Thus, HIV not only exploits DC-SIGN to enhance CD4 + T-cell infection but also circumvents NET-mediated antiviral activity, highlighting its coevolution with the innate immune system [2].

Other viruses also modulate host responses to evade or suppress NETs formation. Feline leukemia virus (FeLV) inhibits neutrophil activation by blocking protein kinase C (PKC) activation, thereby reducing ROS generation. Chronic progressive FeLV infection diminishes neutrophil responsiveness to secondary stimulation, such as that by Leishmania promastigotes, due to prior exhaustive activation, illustrating that viral infection can dampen NETs induction through immune exhaustion [116, 118]. The influenza virus presents another example of NETs involvement in antiviral immunity. It indirectly induces NETs release through influenza-activated lung epithelial cells, which produce superoxide and hydrogen peroxide (H_2_O_2_). In murine models, influenza infection triggers NETs formation in the lungs. However, protein arginine deiminase 4 (PAD4) deficient mice lacking functional NETs did not exhibit increased viral titers or greater susceptibility, suggesting that NETs may not be essential for viral clearance in this context [119, 120]. Despite limited direct evidence, the ability of many viruses to trigger robust neutrophil recruitment suggests that NETs are likely implicated in antiviral defense. NETs have been reported to act against pathogens such as influenza A virus (IAV), HIV, and respiratory syncytial virus (RSV). Mechanistically, NET-associated histones (H3, H4) and antimicrobial peptides (e.g., α-defensin-1, LL-37) exert antiviral effects by neutralizing virions and inhibiting replication. Enzymatic components such as NE and PR3 can degrade viral surface glycoproteins, prevent host cell entry, and limit viral dissemination. Notably, circulating NETs components such as cfDNA, MPO-DNA complexes, and citrullinated histones can serve as potential biomarkers, with elevated plasma levels correlating with disease severity in various viral infections [121, 122].

However, NETs also exhibit pathogenic potential, particularly in the context of hyperinflammation. For instance, in severe acute respiratory syndrome coronavirus 2 (SARS-CoV-2) infection, certain NETs components (e.g., histones) may paradoxically enhance viral infectivity by promoting cell entry [123, 124]. In COVID-19, excessive NETs production has been strongly implicated in immune dysregulation, fueling cytokine storms, acute lung injury (ALI), ARDS, and immunothrombosis. Key NETs components, such as citrullinated histones and cfDNA, contribute to coagulation activation, inhibit fibrinolysis, and exacerbate tissue damage, correlating with worse clinical outcomes. These insights have spurred investigation into NET-targeted interventions, including recombinant DNase therapy, PAD4 inhibitors, and MPO blockade to mitigate inflammation and thrombosis without compromising antiviral immunity [89, 125, 126].

### NETs in autoimmune diseases

**Fig. 3:**
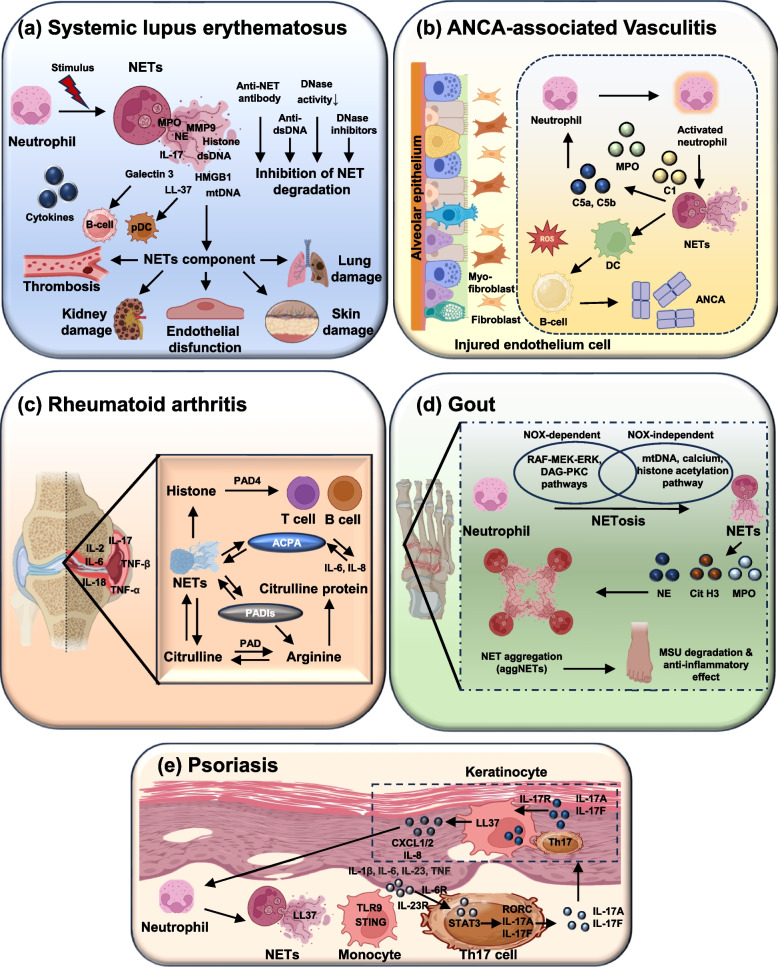
Neutrophil extracellular traps (NETs) in the pathogenesis of autoimmune diseases. This schematic illustrates the involvement of neutrophil extracellular traps in five major autoimmune conditions: **a** systemic lupus erythematosus (SLE): Excessive NETs release combined with impaired clearance due to DNase deficiency, protective factors (C1q, anti-NETs antibodies, anti-dsDNA), or inhibitors promotes NETs persistence. Immunogenic NETs components activate plasmacytoid cells and autoreactive B cells, driving type 1 IFN production, autoantibody generation, immune complex deposition, and tissue injury, particularly in the lung, kidney, skin, and vasculature. **b** ANCA-associated vasculitis (AAV): ANCA antibodies target MPO or PR3 on primed neutrophils, amplifying NETs formation, and promote endothelial injury and fibrosis, contributing to pulmonary and renal pathology. **c** Rheumatoid arthritis (RA): NETs enriched in citrullinated proteins act as sources of autoantigens for ACPA development. PAD4-mediated histone citrullination and autoantibody-driven NETosis create a feedback loop that fuels synovial inflammation, cytokine release (IL-6, IL-8, TNF-α, etc.), and progressive cartilage and bone destruction. **d** Gout: Monosodium urate (MSU) crystal activates the NLRP3 inflammasome, leading to caspase-1 activation and IL-1β/IL-18 release. Recruited neutrophils undergo NETosis, releasing NE, MPO, and citrullinated histones. Aggregated NETs (aggNETs) later exert anti-inflammatory effects by degrading MSU crystals and neutralizing cytokines, balancing inflammation and resolution. **e** Psoriasis: NET-derived DNA and the antimicrobial peptide LL-37 form complexes that activate pDCs via TLR9, inducing IFN-1 and amplifying Th17-driven IL-23/IL-17 signaling, resulting in keratinocyte hyperproliferation. Collectively, the figure emphasizes how dysregulated NETs formation or clearance fuels chronic inflammation and tissue damage across distinct autoimmune settings. Abbreviations: NETs: neutrophil extracellular traps, RA: rheumatoid arthritis, SLE: systemic lupus erythematosus, ACPA: anti-citrullinated protein antibody, PAD4: peptidyl arginine deiminase 4, TNF-α: tumor necrosis factor-alpha, IL-1β: interleukin-1 beta, IL-6: interleukin-6. Note: Figure created using Microsoft PowerPoint and Adobe Illustrator AI. Visual elements were adapted from publicly available scientific graphics and BioRender under fair academic use. No commercial license was obtained

NETs have emerged as pivotal players in the pathophysiology of autoimmune diseases. While NETs are essential for trapping and neutralizing pathogens, their dysregulated formation and impaired clearance can drive chronic inflammation and autoimmunity [127]. In these diseases, NET-derived nuclear and granular components such as DNA, histones, MPO, and NE act as potent autoantigens, triggering the production of pathogenic autoantibodies. This aberrant immune activation sustains a vicious cycle of tissue injury, inflammation, and further NETs release (as shown in Fig. 3).

Systemic Lupus Erythematosus is characterized by systemic inflammation and anti-dsDNA autoantibodies [15]. In SLE, NETosis contributes to autoimmunity by supplying a persistent source of extracellular nuclear material and neutrophil granule proteins that act as autoantigens. Immune complexes containing nucleic acids engage Fcγ receptors on neutrophils and plasmacytoid dendritic cells (pDCs), and trigger TLR7/9 signaling in pDCs to produce type 1 interferons (IFN-1) [74]. INF-α both primes neutrophils for NETs release and drives the expansion of low-density granulocytes (LDGs) that are hyper-NETotic. Key intracellular events include activation of NADPH oxidase (NOX2) and ROS generation, PAD4-mediated histone citrullination, NE/MPO nuclear translocation, and chromatin decondensation [128]. Impaired NETs clearance due to reduced DNase I activity, DNase inhibitors, or anti-DNase/anti-NETs antibodies further prolongs antigen exposure and immune stimulation, creating a self-amplifying loop of NETs release and IFN production. Elevated cfDNA, MPO-DNA complexes, citrullinated histone H3 (H3Cit), nucleosomes, and increased serum NE/MPO activity correlate with SLE activity and lupus nephritis. Autoantibodies that stabilize NETs (anti-DNase, anti-NETs antibodies) and high IFN-signature scores are additional indirect markers of NET-driven pathology [5, 41, 129–131].

ANCA-associated vasculitis is an autoimmune disease in which circulating anti-neutrophil cytoplasmic antibodies (ANCAs) are directed against MPO or PR3, directly activating primed neutrophils via FcγR and complement receptor engagement. This activation triggers NOX2/ROS production, PAD4 activation with histone citrullination, and release of NETs that expose MPO/PR3 as autoantigens, promoting expansion of ANCA responses [132, 133]. NETs deposited on the endothelium promote endothelial injury, complement activation, and thrombogenic cascades, driving necrotizing vasculitis. Neutrophil priming by cytokines (TNF-α, IL-1β) and complement C5a amplifies this process, creating a feed-forward loop between ANCAs, NETosis, and vascular inflammation. Raised MPO-DNA and PR3-DNA complexes, H3Cit, cfDNA, and elevated circulating NE levels are associated with active disease and often fall with immunosuppressive therapy. These markers can reflect disease activity and vascular injury [134–136].

Rheumatoid Arthritis is an autoimmune disease marked by synovial inflammation and the presence of anti-citrullinated protein antibodies (ACPAs). In RA, NETosis contributes to the generation and presentation of citrullinated autoantigens that drive ACPA responses. Synovial neutrophils exposed to cytokines (TNF-α, IL-17A) and immune complexes undergo ROS- and PAD4-dependent NETosis, externalizing citrullinated vimentin, α-enolase, histones, and other antigens [137, 138]. These antigens are taken up by antigen-presenting cells, promoting ACPA production. Stimulus-dependent heterogeneity of NETs composition (for example, NETs containing MMP-8 when induced by RA autoantibodies) may contribute to local tissue destruction and matrix remodeling in joints. The interplay of NETs with synovial fibroblasts and macrophages sustains chronic inflammation and cartilage damage. Increased H3Cit, MPO-DNA complexes, NE activity, nucleosomes, and detection of NET-derived citrullinated proteins in synovial fluid and serum correlate with disease activity and ACPA positivity. Elevated anti-PAD4 autoantibodies are also reported and may reflect PAD4 exposure/immune recognition [9, 127, 139, 140].

Gout flares are triggered by monosodium urate (MSU) crystal deposition, which activates the NLRP3 inflammasome in monocytes/macrophages to release IL-1β. IL-1β induces neutrophil recruitment and IL-8 production, creating a pro-NETotic milieu [141]. MSU crystals directly stimulate neutrophils to release NETs; aggregated NETs (aggNETs) can entrap and degrade proinflammatory mediators, but when opsonization and clearance pathways are suboptimal. NETs' persistence can sustain local inflammation. Importantly, gout-associated NETs are poorly opsonized with complement/CRP and therefore may resist normal scavenging, prolonging inflammation. Local detection of NETs components (cfDNA and H3Cit) in synovial fluid, together with elevated synovial NE/MPO, reflects NETs activity during acute flares. Systemic NETs biomarker data are less established, but cfDNA and MPO-DNA can be transiently raised during severe or prolonged flares [5, 142–144].

Psoriasis is an autoimmune condition in which neutrophils infiltrating the epidermis release NETs enriched in antimicrobial peptides such as LL-37. LL-37 forms complexes with self-DNA that activate pDCs via TLR9, triggering type 1 IFN production and promoting a Th17/Tc17-biased inflammatory response in skin [145]. NETs also provide alarmins that stimulate keratinocyte proliferation and sustain local cytokine production (IL-17, IL-23), perpetuating the psoriatic plaque. The NET-LL-37-DNA axis, therefore, bridges innate NETs formation with adaptive autoimmunity amplification. Elevated lesional and circulating LL-37-DNA complexes, H3Cit, and increased local MPO/NE staining are associated with severity, but biomarkers are primarily useful when combined with clinical scoring [142, 146–148].

### NETs in cardiovascular diseases

**Fig. 4:**
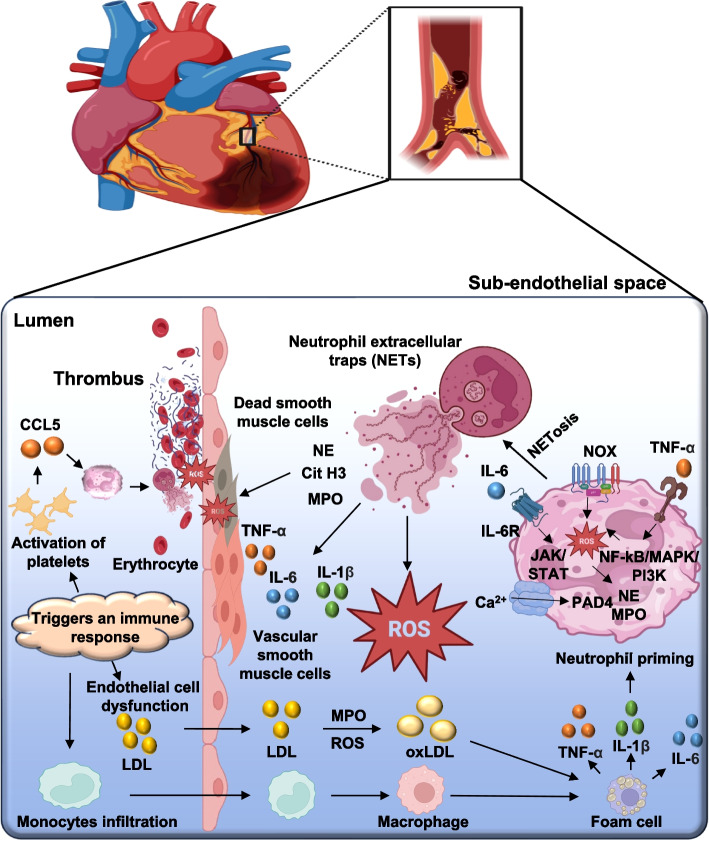
Schematic representation of the role of NETs in atherosclerosis and thrombosis. This figure depicts the involvement of NETs and NETosis in the development and progression of atherosclerosis and thrombotic complications. It shows how the proinflammatory cytokines like TNF-α, IL-1β, and IL-6 produced by ECs, VSMCs, and activated immune cells promote neutrophil activation. Upon stimulation, neutrophils infiltrate atherosclerotic plaques and undergo NETosis, releasing NETs that contribute to plaque destabilization, endothelial damage, platelet activation, and coagulation. The resulting thrombus formation can lead to acute cardiovascular events such as myocardial infarction or stroke. Abbreviations: NETs: neutrophil extracellular traps, TNF-α: tumor necrosis factor-alpha, IL-1β: interleukin-1 beta, IL-6: interleukin-6, ECs: endothelial cells, VSMCs: vascular smooth muscle cells. Note: Figure created using Microsoft PowerPoint and Adobe Illustrator AI. Visual elements were adapted from publicly available scientific graphics and BioRender under fair academic use. No commercial license was obtained

NETs contribute significantly to cardiovascular pathology, particularly in thrombosis and atherosclerosis, by promoting endothelial dysfunction, platelet aggregation, and inflammation [149]. Atherosclerosis is a chronic lipid-driven inflammatory disease characterized by the formation and progression of atherosclerotic plaques, with neutrophils playing a critical pathophysiological role from plaque initiation to rupture. Hyperlipidemia can trigger neutrophilia, and activated neutrophils impair endothelial integrity by depositing granule proteins, enhancing adhesion molecule expression, and recruiting monocytes [150, 151]. Mast cell-derived chemokines such as CXCL1 further promote neutrophil infiltration into the arterial intima. NETs propagate inflammation, promote cytokine release, and destabilize plaques. Pharmacological inhibition of PAD4 reduces both NETs deposition and plaque burden in experimental models, highlighting NETs as a therapeutic target. Neutrophil granule components, such as bactericidal/permeability-increasing protein (BPI), may also participate in plaque-associated thrombosis. Biomarkers, including circulating NETs components (e.g., cfDNA, CitH3) and elevated neutrophil counts, correlate with disease severity (as shown in Fig. 4) [152, 153].

In Acute Coronary Syndrome (ACS), including STEMI, NSTEMI, and unstable angina, neutrophils are involved in early plaque destabilization, myocardial necrosis, and subsequent tissue remodeling. Damage-associated molecular patterns (DAMPs), released from necrotic cardiomyocytes (including HMGB1, mitochondrial DNA, and fibronectin), activate innate immune pathways, such as neutrophil recruitment, adhesion, and trans endothelial migration [154, 155]. Activated neutrophils exacerbate endothelial injury through the induction of apoptosis and the release of proteases, thereby exposing the subendothelial matrix and promoting thrombosis. Thrombin-activated platelets enhance local NETosis, which further activates tissue factor and coagulation cascades. NET-associated alarmins such as S100A8/A9 stimulate TLR4-mediated NLRP3 inflammasome activation, amplifying IL-1/IL-18 production and contributing to myocardial fibrosis. Matrix metalloproteinases (MMP-8, MMP-9) released by neutrophils degrade the extracellular matrix, facilitating adverse cardiac remodeling. Elevated neutrophil count and neutrophil-to-lymphocyte ratio (NLR) are independent prognostic biomarkers in ACS [156–158].

Myocardial Ischemia/Reperfusion Injury (MI/RI), reperfusion following myocardial ischemia is necessary to salvage tissue, but paradoxically induces additional injury through oxidative and inflammatory mechanisms. Within 24–48 h post-reperfusion, neutrophils predominate in the infarcted myocardium, releasing ROS via NADPH oxidative activation [159, 160]. ROS cause direct damage to lipids, proteins, and nucleic acids, while also activating NF-kB-dependent inflammatory gene expression. DAMPs generated during reperfusion activate complement pathways, upregulating endothelial adhesion molecules that promote neutrophil infiltration. Phosphodiesterase 4B (PDE4B)-mediated inflammatory signaling is another driver of neutrophil recruitment and microcirculatory obstruction; PDE4B inhibition improves both myocardial survival and coronary flow in preclinical models. NETs components may serve as mechanistic biomarkers, which include elevated citrullinated histone H3 and cell-free DNA levels correlating with MI/RI severity [161–163].

In hypertension, neutrophil infiltration into vascular, myocardial, and renal tissue contributes to fibrosis and endothelial dysfunction through the release of ROS, pro-inflammatory cytokines, and profibrotic mediators. These processes promote vascular stiffness and maladaptive remodeling. Elevated neutrophil counts and NLR are associated with increased hypertension risk, particularly in non-dipper phenotypes. ROS-driven oxidative stress is a hallmark of neutrophil activation in HTN, suggesting redox-sensitive neutrophil markers could serve as early indicators of vascular injury. Antihypertensive therapies may lower NLR, indicating their impact on systemic inflammatory burden [150, 164–166].

Atrial Fibrillation (AF) is characterized by heightened inflammatory signaling and immune cell infiltration, with neutrophils playing a prominent role in atrial remodeling. Activated neutrophils play a prominent role in atrial remodeling. Activated neutrophils generate platelet-activating factor (PAF) and other lipid mediators, including arrhythmogenic changes in cardiomyocytes [150, 167]. NETs promote cardiomyocyte apoptosis via autophagy-mediated mitochondrial injury, increasing atrial vulnerability. In experimental AF, DNase I-mediated NETs degradation reduces arrhythmia duration and atrial fibrosis, underscoring the pathogenic role of NETs. Clinically, elevated NLR predicts AF recurrence after cardioversion, making it a valuable prognostic biomarker [168, 169].

In heart failure (HF), neutrophils contribute to chronic myocardial injury, maladaptive remodeling, and persistent inflammation. Hemodynamic stress induces the release of cytokines (e.g., TNF-α, IL-1, IL-6), which recruit neutrophils and macrophages [86]. In murine ischemic HF models, depletion of neutrophils attenuates left ventricular remodeling and fibrosis, particularly in the subacute and chronic phases. Neutrophil-derived ROS and proteolytic enzymes directly impair cardiomyocyte contractility. Elevated NLR correlates with worse HF prognosis, making it a simple yet powerful inflammatory biomarker [86, 170, 171].

Viral Myocarditis (VMC), often caused by Coxsackievirus B3 (CVB3), involves both direct viral injury and immune-mediated damage. Viral pathogen-associated molecular patterns (PAMPs) activate TLR signaling in neutrophils, triggering NETs formation [156]. NET-associated proteins, such as S100A8/A9, exacerbate oxidative stress and facilitate viral replication. Neutrophil depletion or PAD4 inhibition reduces myocardial injury and leukocyte infiltration in animal models. NETs markers, including circulating citrullinated histones and myeloperoxidase-DNA complexes, may reflect disease activity and predict disease progression [172–174].

### NETs in cancer

#### Primary tumor progression

NETs are increasingly recognized as active participants in tumor biology, shaping the tumor microenvironment and influencing tumor cell behavior. Tumor-associated neutrophils can acquire either an antitumorigenic N1 or protumorigenic N2 phenotype [59]. In spontaneous intestinal tumor models, low-density neutrophils predominantly polarize toward the N2 subtype and undergo NETosis through complement C3a receptor signaling. Elevated NETs levels have been reported across multiple malignancies, including diffuse large B-cell lymphoma, esophageal adenocarcinoma, lung adenocarcinoma, and gastric cancer, where they correlate with advanced disease stage and poor prognosis. In gastric cancer, NETs have even shown superior prognostic value compared to traditional markers such as carcinoembryonic antigen (CEA) and carbohydrate antigen 19–9 (CA19-9) [175–177].

**Fig. 5:**
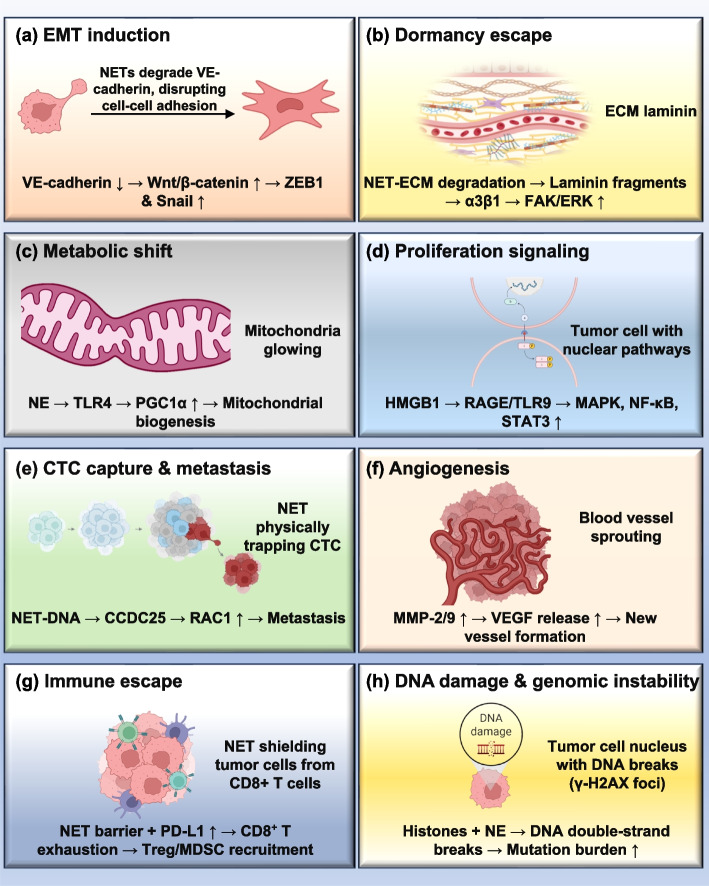
Mechanistic pathways by which neutrophil extracellular traps (NETs) promote tumor progression and metastasis. **a** EMT Induction: NET-induced EMT involves the degradation of VE-cadherin and the activation of the Wnt/β-catenin signaling pathway, as well as the upregulation of ZEB1 and Snail transcription factors. **b** Dormancy Escape: Dormant cancer cells are reactivated by laminin fragments released from NET-mediated ECM degradation, engaging integrin α3β1 and initiating FAK/ERK signaling. **c** Metabolic Shift: NET-mediated NE activates TLR4, inducing PGC1α-mediated mitochondrial biogenesis and metabolic adaptation. **d** Proliferation Signaling: HMGB1 released from NETs binds to RAGE and TLR9, triggering MAPK, NF-kB, and STAT3 pathways that drive proliferation. **e** CTC capture & Metastasis: CTCs are physically trapped by NET-DNA and engage CCDC25 receptors, activating RAC1 signaling and promoting metastasis. **f** Angiogenesis: NETs upregulated MMP-2 and MMP-9, facilitating VEGF release and angiogenesis. **g** Immune Escape: NETs enhance immune evasion by shielding tumor cells, promoting PD-L1 expression, inducing CD8 + T cell exhaustion, and recruiting Tregs and MDSCs. **h** DNA damage & Genomic instability: NET-associated components, such as histones and NE, induce DNA damage within tumor cells. These are evidenced by γ-H2AX foci and cause double-strand chromosomal breaks, leading to genomic instability, enhanced mutation burden, and potential therapy resistance. Abbreviations: NETs: neutrophil extracellular traps, EMT: epithelial-mesenchymal transition, NE: neutrophil elastase, MMP: matrix metalloproteinase, PGC1α: peroxisome proliferator-activated receptor gamma coactivator 1-alpha, HMGB1: high mobility group box 1, RAGE: receptor for advanced glycation end-products, TLR: toll-like receptor, CTC: circulating tumor cell, CCDC25: coiled-coil domain-containing protein 25, FAK: focal adhesion kinase, VEGF: vascular endothelial growth factor, PD-L1: programmed death-ligand 1, Treg: regulatory T cell, MDSC: myeloid-derived suppressor cell. Note: Figure created using Microsoft PowerPoint and Adobe Illustrator AI. Visual elements were adapted from publicly available scientific graphics and BioRender under fair academic use. No commercial license was obtained

Mechanistically, NETs support tumor progression through activation of oncogenic signaling cascades. In hepatocellular carcinoma, inhibition of NETs formation reduces tumor growth in non-alcoholic steatohepatitis models. In glioma and colorectal cancer, NETs drive proliferation via NF-kB, STAT3, and p38 MAPK pathways [178, 179]. NET-derived HMGB1 further promotes tumor growth by activating RAGE and TLR9, leading to MAP kinase and NF-kB signaling. In pancreatic cancer, NETs' DNA stimulates pancreatic stellate cells via RAGE, fueling desmoplasia and tumor expansion. Neutrophil elastase contributes by upregulating mitochondrial biogenesis through the TLR4-PGC1α axis, enhancing metabolic adaptation in colorectal and hepatocellular carcinomas (as shown in Fig. 5) [180–182].

#### Metastasis and organotropism

**Fig. 6:**
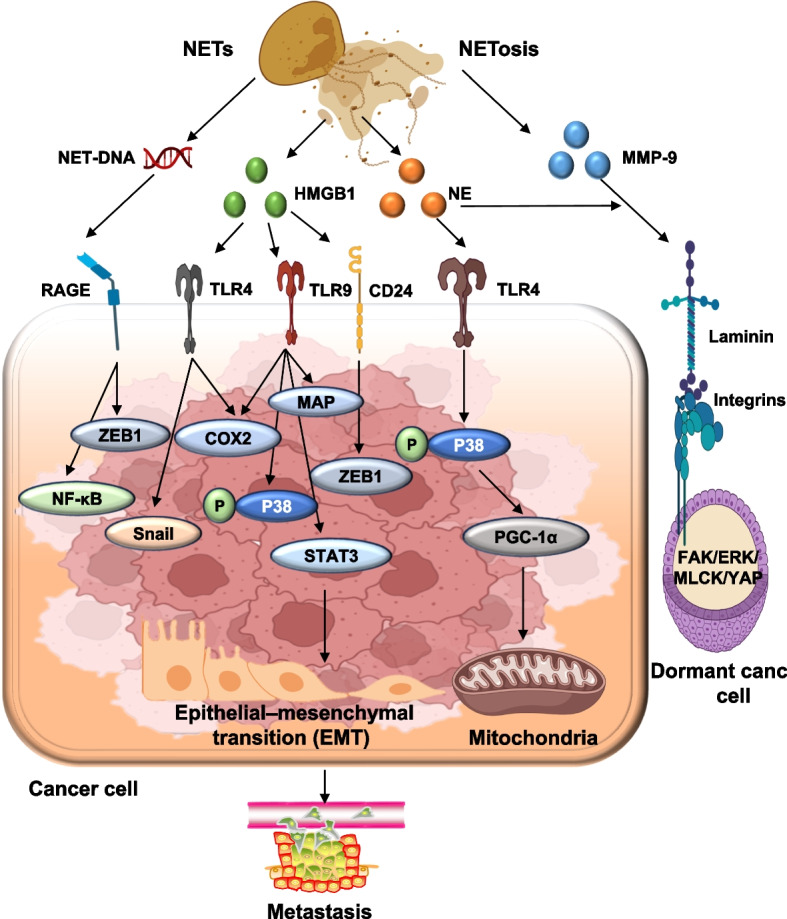
NET-mediated mechanisms of tumor progression and metastasis. This figure illustrates the multifactorial role of NETs in promoting tumor proliferation, metabolic reprogramming, reactivation of dormant cancer cells, and metastatic spread. The schematic highlights how NET-associated HMGB1 interacts with receptors such as TLR2, TLR4, TLR9, CD24, and RAGE to activate oncogenic signaling pathways, including STAT3, p38 MAPK, and NF-kB. NE enhances tumor cell metabolism through the TLR4-PGC1-α axis. MMP-9 and NE degrade laminin in the extracellular matrix, triggering integrin α3β1-mediated signaling cascades involving FAK, ERK, MLCK, and YAP, which facilitate reactivation of dormant cancer cells. Additionally, NET-derived DNA activates pancreatic stellate cells via RAGE, promoting pancreatic tumor growth, and influences gastric cancer progression via BRF1 signaling. The figure also depicts NET-mediated enhancement of EMT through upregulation of transcription factors such as ZEB1 and Snail, along with modulation of COX-2 and MAPK pathways that support tumor cell migration and inflammatory signaling. Abbreviations: NETs: neutrophil extracellular traps, HMGB1: high mobility group box 1, TLR: toll-like receptor, RAGE: receptor for advanced glycation end products, STAT3: signal transducer and activator of transcription 3, MAPK: mitogen-activated protein kinase, NF-kB: nuclear factor Kappa B, NE: neutrophil elastase, PGC1-α: peroxisome proliferator-activated receptor gamma coactivator 1-alpha, MMP-9: Matrix metalloproteinase-9, FAK: focal adhesion kinase, ERK: extracellular signal-regulated kinase, MLCK: myosin light chain kinase, YAP: Yes-associated protein, EMT: epithelial-to mesenchymal transition, COX-2: cyclooxygenase-2, ZEB1: zinc finger E-box-binding homeobox 1. Note: Figure created using Microsoft PowerPoint and Adobe Illustrator AI. Visual elements were adapted from publicly available scientific graphics and BioRender under fair academic use. No commercial license was obtained

NETs also play a critical role in metastatic dissemination. By remodeling the extracellular matrix (ECM) and activating integrin-dependent pathways, NETs enhance tumor cell adhesion, migration, and invasion. In breast cancer, NETs' DNA binds laminin within the ECM, triggering integrin α3β1/FAK/ERK/MLCK/YAP signaling that reactivates dormant tumor cells [183, 184]. Concurrently, degradation of ECM inhibitors such as thrombospondin-1 removes natural barriers to dissemination. In systemic inflammatory conditions such as sepsis, NETs physically trap circulating tumor cells through β1-integrin interactions, thereby increasing metastatic efficiency. Organ-specific accumulation of NETs also promotes metastasis: in ovarian cancer, NETs localize to the omentum, a premetastatic niche. Where NET-derived HMGB1 activates TLR9-MAPK signaling. NET-induced epithelial-to-mesenchymal transition (EMT) has been observed in breast and gastric cancers, further increasing invasiveness. In addition, NETs exposure upregulates cyclooxygenase-2 (COX-2), sustaining a proinflammatory tumor microenvironment that favors metastatic outgrowth (as shown in Fig. 6) [185–187].

#### Therapy resistance

Emerging evidence implicates NETs in therapy resistance, particularly in the context of immune checkpoint inhibitors (ICIs). Elevated circulating levels of citrullinated histone H3, a surrogate marker of NETs, are associated with poorer overall and progression-free survival in patients with advanced non-small cell lung cancer treated with anti-PD-1/PD-L1 therapy [188–190]. NETs contribute to immune evasion by promoting T-cell exhaustion, decorating tumor and immune cells with PD-L1, and activating IL-17-driven pathways that impair CD8 + T cell and NK cell cytotoxicity. For example, IL-17-induced NETosis in pancreatic ductal adenocarcinoma blunts the efficacy of anti-PD-1 therapy. NET-associated HMGB1 further amplifies immunosuppression via RAGE/TLR9 signaling [191, 192].

NET-related gene signatures and integrated biomarker panels, including NETs markers, IL-8, and C-reactive proteins, are now under development to predict ICI response. Preclinical models demonstrate that pharmacological disruption of NETs, using DNase I or PAD4 inhibitors, can restore sensitivity to checkpoint blockade in colorectal and pancreatic cancers. These findings suggest that targeting NETs in combination with ICIs may represent a promising therapeutic strategy to overcome resistance in multiple tumor types [188, 193, 194].

### Other related diseases

Asthma has traditionally been linked to eosinophilic inflammation, and neutrophils and NETs are now recognized as key contributors, particularly in severe and steroid-resistant forms [195]. NETs have been detected in bronchoalveolar lavage fluid of patients with allergic asthma, where increased IL-8 levels and neutrophil counts distinguish severe from moderate disease. IL-8 not only drives neutrophil recruitment but also directly induces NETosis. Platelet activation, which is enhanced in asthma, further amplifies NETs formation, thereby reinforcing airway inflammation and tissue injury [196–198].

In Alzheimer’s disease (AD), NETs play a central role in neuroinflammation and vascular dysfunction. Amyloid-β, inflammatory cytokines, and activated platelets can all trigger NETosis within the cerebral vasculature [199, 200]. NETs components, including extracellular DNA, histones, neutrophil elastase, and matrix metalloproteinases, damage the blood–brain barrier by degrading tight junction proteins and activating endothelial cells through β2 integrins. These processes promote thromboinflammation, microvascular occlusion, and reduced cerebral perfusion. Elevated proinflammatory mediators such as IL-1β, IL-8, TNF-α, and HMGB1 sustain NETs activity through RAGE- and TLR4-dependent pathways. Collectively, these mechanisms link NETs to chronic neuroinflammation and progressive vascular injury in AD [50, 87, 201, 202].

Hyperglycemia in both type 1 and type 2 diabetes primes neutrophils for excessive NETosis by enhancing oxidative stress. In type 1 diabetes, elevated circulating NET-associated proteins such as proteinase 3, neutrophil elastase, and MPO-DNA complexes appear early in disease progression [203, 204]. In type 2 diabetes, biomarkers including cell-free DNA, nucleosomes, and neutrophil elastase are consistently increased. Functionally, NETs impair wound healing, exacerbate neuropathy, nephropathy, and retinopathy, and promote tissue inflammation. PAD4 overexpression and histone citrullination further disrupt repair processes, while pharmacological inhibition of PAD4 or treatment with DNase restores healing and reduces inflammatory burden [205–207].

Pregnancy is characterized by distinct immunological adaptations, including elevated white blood cell counts and a mild neutrophilia. Studies have shown that neutrophils in pregnant women display heightened activation and an increased capacity for phagocytosis compared with those in non-pregnant women [208, 209]. Despite these observations, the mechanisms driving enhanced neutrophil activity during pregnancy remain incompletely understood. One of the most critical pregnancy complications is pre-eclampsia (PE), a leading contributor to maternal morbidity and mortality worldwide [210, 211]. In PE, leukocyte activation, particularly of neutrophils, is markedly increased. Evidence implicates NETs in the pathogenesis of PE, with histological studies identifying NETs in placental tissue near trophoblasts. Elevated NETs levels in the placental intervillous space, together with increased maternal cfDNA, correlate with disease severity. NETs are now recognized as a principal source of cfDNA in maternal plasma during PE [212–214]. Experimental studies further demonstrate that placental fragments, syncytiotrophoblast particles, and endothelial-derived factors stimulate NETs release, while DNA from damaged placental cells amplifies this process. The resulting positive feedback loop contributes to endothelial damage, coagulation abnormalities, and heightened thrombotic risk. In addition, placental NETs may provoke autoimmune responses, although it remains unclear whether they are primary drivers of pathology or secondary to placental dysfunction [215, 216]. Gestational diabetes mellitus (GDM), a transient state of glucose intolerance during pregnancy, also shows a strong connection to NETs activity [217]. Pregnancies complicated by GDM are at increased risk of developing PE. Circulating neutrophils in GDM exhibit an exaggerated pro-NETotic phenotype, characterized by spontaneous NETs formation and increased placental infiltration, particularly with NE. NE disrupts trophoblast physiology and alters glucose metabolism through the modulation of key signaling pathways [218–220]. Moreover, hypoadiponectinemia in GDM has been identified as a trigger for NETs formation, promoting trophoblast apoptosis via reactive oxygen species-dependent mitochondrial activation and ERK1/2 signaling. Importantly, pharmacological inhibition of TNF-α with infliximab reduces the pro-NETotic effect of GDM sera in vitro, suggesting a potential therapeutic avenue. Supporting this, NET-deficient PAD4-/- mice with increased GDM display increased placental weight compared with wild-type controls, further underscoring the role of altered NETs activity in the development of GDM-associated complications [220–223]. NETs have also been implicated in pregnancy loss and spontaneous abortion [224]. Elevated fetal cfDNA levels in maternal circulation have been associated with spontaneous abortion, while dysregulated low-density neutrophils (LDNs) show increased NETs formation. NETs have been detected in placental tissues from women experiencing miscarriage, along with elevated levels of MPO and pentraxin 3. Similarly, increased chroioamniotic NETs deposition has been reported in cases of chrioamnionitis and preterm delivery. In experimental models, PAD4-deficient mice exhibit significantly reduced inflammatory and thrombotic responses, leading to fewer pregnancy losses [225–228]. These findings suggest that targeting NETs formation could represent a promising therapeutic strategy for pregnancy disorders linked to impaired placentation and inflammation.

## Detection of neutrophil NETs

Accurate detection of NETs is fundamental for understanding their roles in physiology and pathology. A wide spectrum of classical and modern approaches has been developed, each with distinct strengths and limitations regarding sensitivity, specificity, and suitability for different sample types. The integration of imaging, biochemical, and molecular assays has greatly advanced our ability to study NETs biology both in vitro and in vivo [229].

### Classical methods

Early efforts to study NETs relied on imaging and antibody-based approaches, which remain foundational for the field. These techniques enable visualization and quantification of NETs structures, though they vary in resolution, scalability, and specificity. The earliest and most widely adopted approach for NETs detection is immunofluorescence microscopy, which remains the gold standard. This technique relies on the co-localization of extracellular DNA with NET-associated proteins such as NE, MPO, and citrullinated histone H3 (citH3) [230]. By combining DNA dyes (e.g., DAPI, SYTOX) with antibody staining, microscopy allows direct visualization of NETs structures and their spatial relationship with surrounding cells or tissues. More advanced modalities, including scanning and transmission electron microscopy with immune-gold labeling, have provided ultrastructural insights into NETs' architecture. However, the reliability of these ultrastructural techniques depends heavily on meticulous sample preparation and fixation, which can introduce artifacts and limit throughput [231, 232].

To complement visual, enzyme-linked immunosorbent assays (ELISAs) have been developed for quantitative measurement of NETs components in biological fluids such as plasma, serum, or bronchoalveolar lavage. These assays typically detect complexes of DNA with NE, MPO, or citH3 using antibody-based capture and chromogenic substrates [233, 234]. Their high-throughput nature makes them well-suited for large clinical studies, particularly in conditions such as sepsis, autoimmune disease, and cancer. Nonetheless, the absence of spatial resolution and the potential for cross-reactivity with non-NET-derived molecules are major limitations. Consequently, ELISA measurements are often paired with microscopy-based validation to ensure specificity [235, 236].

### Modern molecular approaches

With technological advances, newer molecular and cytometric platforms have emerged that provide higher resolution and functional insights. These approaches capture the heterogeneity of NETosis and complement classical assays by linking NETs dynamics to transcriptional and cellular states. Recent advances have introduced molecular techniques that capture the dynamic and heterogeneous nature of NETosis. An emerging tool is single-cell RNA sequencing (scRNA-seq), which enables the profiling of gene expression in individual neutrophils undergoing NETosis. This technique has uncovered transcriptional signatures associated with NETs formation, including pathways linked to reactive oxygen species generation, autophagy, and chromatin remodeling. By highlighting the cellular heterogeneity of neutrophil responses, scRNA-seq has expanded our understanding of NETs regulation beyond bulk population analyses. Although not yet widely applied in clinical practice, single-cell approaches hold significant promise for unraveling patient-specific differences in NETs biology, particularly in complex diseases such as autoimmune disorders and cancer [237, 238].

Another emerging technique is flow cytometry, which provides a high-throughput and sensitive approach to detect NET-related markers at the single-cell level. Using fluorophore-conjugated antibodies against MPO, citH3, or NE in combination with DNA-binding dyes such as SYTOX Green, flow cytometry can distinguish between NETosis, apoptosis, and other neutrophil activation states. This ability to analyze large cell populations quantitatively makes flow cytometry especially valuable in translational studies and clinical trials [239–241]. Together, these complementary methods form a robust toolkit for studying NETs. Microscopy remains indispensable for structural validation, ELSIA offers scalable quantification, flow cytometry provides population-level sensitivity, and single-cell sequencing reveals regulatory complexity. The continued refinement and integration of these techniques will be critical for translating NETs research into diagnostic and therapeutic applications.

## Therapeutic strategies for targeting NETs

**Fig. 7:**
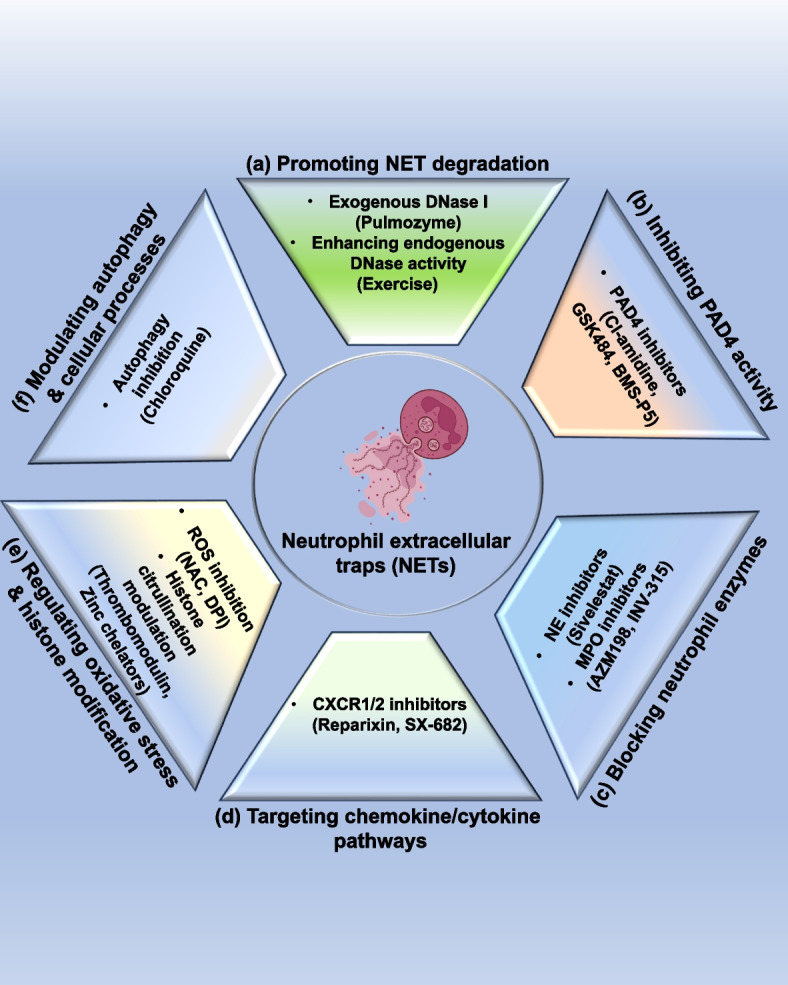
Therapeutic strategies targeting neutrophil extracellular traps (NETs). This schematic illustrates pharmacological and biological strategies for modulating NETs biology. At the core, neutrophils undergo NETosis, releasing extracellular DNA fibers decorated with granule proteins. Therapeutic interventions can be grouped into six categories: **a** Promoting NETs degradation (DNase I therapy, enhanced endogenous DNase activity), **b** Inhibiting PAD4 activity (CI-amidine, GSK484, BMS-P5), **c** Blocking neutrophil enzymes (NE and MPO inhibitors), **d** Targeting chemokine/cytokine pathways (CXCR1/2 blockade), **e** Regulating oxidative stress and histone modification (ROS inhibitors, citrullination modulators), and (**f**) Modulating autophagy and cellular processes (chloroquine). Collectively, these approaches aim to suppress NET-driven tumor progression, inflammation, and immune evasion. Abbreviations: CXCR1/2: C-X-C chemokine receptor type 1/2, DNase I: Deoxyribonuclease I, MPO: myeloperoxidase, NE: neutrophil extracellular traps, PAD4: Peptidyl arginine deiminase 4, ROS: reactive oxygen species. Note: Figure created using Microsoft PowerPoint and Adobe Illustrator AI. Visual elements were adapted from publicly available scientific graphics and BioRender under fair academic use. No commercial license was obtained

Aberrant or excessive NETs formation has emerged as a critical contributor to the development of autoimmune disorders, cancer progression, and thrombo-inflammatory complications. Current therapeutic strategies can be broadly divided into three categories: (i) suppression of NETs formation, (ii) facilitation of NETs degradation, and (iii) modulation of their release (as shown in Fig. 7). These interventions aim to counteract the pathological effects of NETs while safeguarding their protective role in innate immunity. As outlined in Table 2, several pharmacological inhibitors have been developed to modulate NET-inducing pathways, offering potential therapeutic avenues in inflammatory, autoimmune, and malignant disorders.

**Table 2:** Experimental pharmacological inhibitors targeting NETosis pathways

Target/Pathway	Pharmacological agent	Disease model/ Experimental context	Mechanism of action	Key preclinical findings	Refs
**Targeting NETs formation**					
PAD4 (Histone citrullination)	CI-amidine	Rheumatoid arthritis	Inhibits PAD4-mediated histone citrullination, blocking chromatin decondensation	Decreased NETs formation, reduced joint inflammation, and edema	[242]
		Inflammatory bowel disease	Suppresses tissue NETosis	Ameliorated mucosal inflammation	[243]
		Type 1 diabetes	Prevents exposure of citrullinated autoantigens	Reduces autoantibody generation	[244]
		Pulmonary cancer	Inhibits NETs-driven metastatic seeding	Decreased pulmonary metastatic burden	[245]
	BB-CI-amidine	SLE model	PAD4 inhibition	Attenuated proteinuria and immune complex deposition	[246]
		Colon cancer	Blocks NETs deposition in the metastatic niche	Reduces liver metastases	[247]
	GSK484	Breast cancer	Selective PAD4 inhibitor	Decreased lung metastasis via NETs suppression	[40]
		Ovarian cancer	Inhibits PAD4-dependent NETs	Reduced omental colonization	[248]
	BMS-P5	Multiple myeloma	PAD4 inhibition	Prolongs animal survival, reduces NETosis	[249]
Neutrophil elastase (NE)	Sivelestat	Atherosclerosis	Inhibit NE-dependent chromatin decondensation	Lowered lipid accumulation and vascular inflammation	[250]
		Inflammatory bowel disease	Suppresses NE-mediated NETs release	Decreased colitis severity	[251]
		Psoriasis	Reduces NE-driven epidermal inflammation	Diminished T-cell infiltration	[252]
		Colon cancer	Inhibits NE-induced NETs scaffolds	Decreased hepatic and pulmonary colonization	[253]
	GW311616A	Lung cancer	Potent NE inhibitor	Reduced hepatic metastasis	[254]
	CHF6333	Bronchiectasis	NE inhibition	Decreases airway infection and inflammation	[255]
Myeloperoxidase (MPO)	PF-1355	Glomerulonephritis	Inhibit MPO catalytic activity	Improved renal histology and reduced chronic injury	[256]
	INV-315	Atherosclerosis	MPO inhibition	Reduced plaque area and improved endothelial function	[257]
	AZM198	Crescentic glomerulonephritis	MPO inhibition	Protected endothelial integrity	[85]
	NBD peptide	Breast cancer	Inhibits the MPO-NETs pathway	Reduced tumor growth	[258]
CXCR1/2 signaling	Reparixin	Breast cancer	Blocks CXCL8-CXCR1/2 axis	Enhanced efficacy of the immune-checkpoint therapy	[259]
	AZD5069	COPD/asthma	CXCR2 antagonist	Reduces neutrophil recruitment and airway inflammation	[260]
Autophagy/ PI3K signaling	Chloroquine	Cancer-related thrombosis	Inhibits autophagy-mediated NETosis	Decreased perioperative thrombosis	[261]
	Wortmannin	Acute promyelocytic leukemia	PI3K/autophagy inhibition	Reduces LC3 aggregation and extracellular trap formation	[262]
**Targeting NETs depletion**					
NETs clearance (DNA degradation)	DNase I	Colitis	Digests extracellular DNA scaffolds	Attenuates colitis and tumorigenesis	[263]
		Lung cancer	Degrades NETs structures	Reduces hepatic adhesion and metastasis	[264]
		Breast cancer	Degrades circulating NETs	Decreased lung metastasis and thrombosis	[265, 266]
		Pancreatic cancer	DNase-mediated DNA degradation	Reduces liver metastases	[267]
**Targeting NETs release**					
ROS	N-acetyl-L-cysteine (NAC)	Biliary atresia	Antioxidant suppresses ROS accumulation	Delayed disease onset reduces mortality	[268]
	Diphenyleneiodonium (DPI)	Sepsis model	NADPH oxidase inhibition	Reduces cytotoxicity and NETs formation	[269]
	AZD7986	Breast cancer	Inhibits ROS-dependent NETs	Reduced pulmonary metastasis	[270]
Citrullinated histone H3 (citH3)	Recombinant thrombomodulin	Sepsis	Binds CiH3, limits NET-induced toxicity	Reduces AKI, lung injury	[271]
Zinc homeostasis	Zinc supplementation	Zinc deficiency model	Cofactor restoration for immune defense	Maintains innate immune competence and reduces NETs dysregulation	[272]

*Abbreviations: AKI* Acute Kidney Injury, *APL* Acute promyelocytic leukemia, *APM* antigen presentation machinery, *ATP* adenosine triphosphate, *AZD7986* cathepsin C inhibitor (dipeptidyl peptidase 1 inhibitor), *BB-CI-amidine* a chlorinated derivative of CI-amidine (PAD4 inhibitors), *BMS-P5* selective PAD4 inhibitor, *CHF6333* oral neutrophil elastase inhibitor, *CitH3* citrullinated histone H3, *CI-amidine* Pan-Pad inhibitor, *COPD* chronic obstructive pulmonary disease, *CXCR1/2* CXC chemokine receptor 1/2, *DAMPs* damage-associated molecular patterns, *DNA* deoxyribonucleic acid, *DNase I* deoxyribonuclease I, *DNP* dinitrophenol (ROS inhibitors), *DPI* diphenyleneiodonium (NADPH oxidase inhibitors), *ET* extracellular trap, *GSK484* selective PAD4 inhibitor, *GW311616A* orally bioavailable NE inhibitor, *ICI* immune checkpoint inhibitor, *IBD* inflammatory bowel disease, *IL-8* interleukin-8, *INV-315* MPO inhibitor with anti-atherosclerotic activity, *LC3* microtubule-associated protein 1A/1B-light chain 3, *MPO* myeloperoxidase, *NAC* N-acetylcysteine, *NE* neutrophil elastase, *NETs* neutrophil extracellular traps, *PAD4* peptidyl arginine deiminase-4, *PF-1355* MPO inhibitor, *PI3K* phosphoinositide 3-kinase, *ROS* reactive oxygen species, *SLE* systemic lupus erythematosus, *Sivelestat* specific neutrophil elastase inhibitor, *VCAM* vascular cell adhesion molecule, *Wortmannin* PI3K inhibitor

### Inhibition of NETs formation

The most direct therapeutic approach targets the upstream molecular machinery of NETosis. By blocking critical enzymes and signaling pathways involved in chromatin decondensation and nuclear rupture, these strategies aim to suppress aberrant NETs release at their origin while sparing essential antimicrobial functions. First, protein PAD4-mediated histone citrullination is essential for chromatin relaxation during NETosis. Small molecule PAD4 inhibitors, including CI-amidine and GSK484, effectively suppress chromatin decondensation, thereby reducing inflammatory signaling, autoimmunity progression, and tumor dissemination in preclinical studies. BMS-P5, a recently developed PAD4 inhibitor, exhibits enhanced potency, isoform selectivity, and oral bioavailability with minimal off-target effects, making it a leading candidate for clinical evaluation [246]. Despite these advances, unresolved challenges remain regarding isoform-specific targeting and the risk of immune perturbation, underscoring the importance of further structural optimization [54, 273, 274]. Secondly, NE drives chromatin decondensation and nuclear envelope rupture during NETs formation. Pharmacological blockade with clinically available agents such as Sivelestat has proven effective in mitigating NET-mediated tissue damage in ARDS and sepsis [250]. Next-generation NE inhibitors, including GW311616A and CHF6333, show improved pharmacokinetic properties and exhibit antitumor efficacy, and concerns regarding chronic administration remain barriers to widespread therapeutic application [253, 275]. Third, MPO facilitates the oxidative reactions necessary for NETs release. Inhibitors such as AZM198 (irreversible) and INV-315 (reversible) demonstrate efficacy in reducing NET-dependent vascular inflammation and atherosclerotic burden [85, 257]. Nevertheless, clinical translation has been hindered by systemic toxicities such as PF-1355-induced tachycardia and an incomplete understanding of redox-mediated regulatory pathways [256]. Furthermore, antibody-mediated neutralization of NETs components represents another mechanistic avenue of intervention. Monoclonal antibodies directed against citrullinated histone H3, NE, and MPO have been shown to disrupt NETs scaffolds, thereby limiting pro-thrombotic and pro-inflammatory signaling cascades [276, 277]. Recombinant human thrombomodulin not only suppresses NETs release but also neutralizes extracellular histones, reducing endothelial injury and organ dysfunction in experimental models. These findings highlight antibody-based strategies as precision tools for dismantling pathogenic NETs while maintaining immune surveillance [278].

On the other hand, cytokines and cellular physiological processes significantly influence NETs formation. It has been reported that chemokine receptors CXCR1 and CXCR2 regulate neutrophil recruitment to inflammatory sites and tumor microenvironments. Antagonists such as Reparixin and SX-682 effectively block these pathways, reducing excessive neutrophil infiltration and suppressing the activity of myeloid-derived suppressor cells (MDSCs). Preclinical studies suggest that CXCR1/2 blockade not only alleviates chronic inflammation but also enhances antitumor immunity. Ongoing clinical trials are now assessing the synergistic potential of these agents when combined with immune checkpoint inhibitors in metastatic malignancies [259, 260]. Autophagy has emerged as a pivotal intracellular process influencing the dynamics of NETs formation. Mechanistically, autophagy flux regulates chromatin decondensation and the trafficking of granular enzymes during NETosis. Inhibition of autophagy with pharmacological agents such as chloroquine suppresses PAD4-mediated histone citrullination and LC3-II accumulation, thereby attenuating NETs release. Preclinical studies indicate that autophagy blockade reduces NET-driven thrombosis and inflammatory injury in models of vasculitis and cancer. However, autophagy is essential for cellular homeostasis, metabolic adaptation, and pathogen clearance. Therapeutic strategies must carefully balance the suppression of aberrant NETs formation with the preservation of protective autophagic functions [261, 262].

### Promotion of NETs degradation and clearance

An alternative strategy focuses on dismantling or eliminating NETs once they are formed. This involves the enzymatic degradation of extracellular DNA, enhancement of endogenous nuclease activity, and facilitation of phagocytic clearance, thereby restoring tissue homeostasis and limiting immunopathology. One of the most direct approaches to dismantling NETs is the enzymatic degradation of their DNA backbone. Recombinant DNase I effectively cleaves extracellular chromatin, diminishing NET-mediated vascular occlusion and tissue injury. In experimental models, DNase I reduces inflammation in rheumatoid arthritis and colitis, although its efficacy is reduced in systemic lupus erythematosus (SLE) due to the presence of NET-stabilizing proteins. Beyond inflammation, DNase I interferes with tumor-endothelium adhesion, thereby exerting anti-metastatic effects. The clinical analog, Pulmozyme, originally developed for cystic fibrosis, is now being repurposed for indications such as cancer-associated thrombosis and metastasis, underscoring its translational potential [9, 279, 280].

Augmenting intrinsic DNase activity provides an alternative strategy to accelerate NETs degradation. Emerging evidence shows that physiological interventions, including sustained physical activity, reduce circulating cell-free DNA and enhance DNase function. This suggests that lifestyle-associated modulation of nuclease activity could represent a complementary, non-pharmacological means of promoting NETs clearance, particularly in chronic inflammatory states [264]. Additionally, phagocytic clearance represents a physiological route of NETs resolution. Macrophages and dendritic cells internalize and enzymatically degrade the NETs structure, thereby restoring tissue homeostasis. Therapeutic approaches aimed at enhancing these functions, for instance, by boosting efferocytosis, may accelerate NETs clearance [281, 282]. Additionally, activated protein C (APC) facilitates NETs resolution by cleaving histones and disrupting neutrophil-platelet aggregates, providing a mechanistic link between coagulation control and immunoregulation [283].

### Regulation NETs release

Rather than complete inhibition or degradation, regulatory approaches fine-tune the signaling cascades that govern NETs' extrusion. By modulating oxidative stress, histone modification, and iron-dependent pathways, these interventions seek to recalibrate NETs' dynamics to reduce pathological effects without compromising host defense. Reactive oxygen species (ROS) are key mediators of chromatin decondensation and NETs extrusion. Pharmacological antioxidants such as N-acetylcysteine (NAC) and NADPH oxidase inhibitors (e.g., diphenyleneiodonium, DPI) mitigate NET-driven injury by dampening ROS signaling. Inhibition of cathepsin C (e.g., AZD7986) further limits protease activation, reducing tissue damage in sepsis and cancer models. Notably, combinatorial approaches such as exenatide (a GLP-1 receptor agonist) combined with immune checkpoint blockade, enhance antitumor immunity by counteracting NET-mediated immunosuppression, illustrating how ROS modulation can synergize with existing therapies [268, 269].

Histone citrullination, catalyzed by PAD4, is a central driver of chromatin decondensation during NETosis. Therapeutic interventions targeting this modification reduce the pro-thrombotic and pro-inflammatory potential of NETs. For example, thrombomodulin and zinc chelators inhibit citrullinated histone H3 activity, thereby reducing organ damage in experimental sepsis and pancreatic cancer models. Interestingly, zinc supplementation has been reported to exert context-dependent effects; while excessive zinc promotes NETs release, physiologic levels appear to stabilize NETs structure for antimicrobial defense while limiting immunothrombotic complications. This dual role highlights the complexity of ion-dependent regulation in NETs biology [272].

### Clinical translation

The therapeutic and diagnostic potential of NETs modulation is now being explored across diverse clinical contexts. In cancer, NET-related proteins correlate with disease progression and treatment resistance, suggesting their use as biomarkers for prognosis and therapeutic response. In juvenile idiopathic arthritis, NETs levels track with disease activity, and TNF-α inhibitors have been shown to suppress the formation [284]. Table 3 provides an overview of experimental and clinical studies investigating the pathogenic and mechanistic roles of NETs across infectious, autoimmune, cardiovascular, and neoplastic diseases.

**Table 3:** Experimental and preclinical studies demonstrating the role of NETs in pathogen-associated conditions or disease pathogenesis

Pathogens/Disease	Year	Model type	Key findings	Pathophysiological implications	Refs
**Pathogen-associated conditions**					
*Leishmania amazonensis*	2009	In vitro/mouse	Promastigotes stimulate NETs release, reducing parasite survival	NETs act as an early innate defense mechanism	[285]
*Leishmania donovani*	2010	In vitro	Promastigotes evade NETs killing via nucleases	Identifies parasite immune evasion strategy	[286]
*Leishmania spp.*	2014	Human neutrophils	Parasite nucleases degrade the NETs DNA scaffold	Targeting nuclease activity may restore NETs defense	[287]
*Leishmania spp.*	2015	Human neutrophils	ROS-dependent and ROS-independent NETs formation	Dual-pathway activation highlights complex host–pathogen dynamics and therapeutic opportunities	[288]
*Eimeria bovis*	2010	Bovine neutrophils & animal model	NETs form as part of the innate immune response against protozoal invasion	Confirms NETs' defense across species, supporting translational relevance in zoonotic infections	[289]
*Toxoplasma gondii*	2012	Human & mouse neutrophils	NETs restrict parasite spread in both species	Demonstrates evolutionary conservation of NET-mediated defense, relevant for congenital toxoplasmosis	[290]
*Strongyloides stercoralis*	2014	Human & mouse neutrophils/macrophages	NETs cooperate with macrophages to eliminate larvae	Highlights NET-macrophage synergy as a novel therapeutic target in helminth infections	[291]
Pro-inflammatory role (pathogen-associated)	2021	Human neutrophils	NETs amplify cytokine release and oxidative burst, sustaining inflammation	Suggests NETs contribute to chronic inflammatory pathology and could be biomarkers for disease progression	[83]
**Disease pathogenesis**					
Atherosclerosis	2014	Mouse model	PAD4 inhibition reduced NETs formation, lowering plaque burden and arterial thrombosis	NET-targeted therapies may offer cardiovascular protection by limiting thrombosis	[292]
	2015	Mouse model	Cholesterol crystals triggered NETs release; NETs primed macrophages for cytokine production. Blocking NETs reduced plaque progression	Links sterile crystal deposition with inflammation, positioning NETs as a therapeutic target in early atherogenesis	[149]
	2015	Human carotid plaque analysis (*n* = 56)	NETs detected within plaques, associated with luminal apoptotic endothelial cells	Confirms translational relevance of NETs in human atherosclerotic pathology	[293]
	2015	STEMI patients (In vitro)	Neutrophils from infract-related arteries showed enhanced NETs formation vs non-infract regions	Suggests NETs contribute to acute coronary thrombosis, not just chronic plaque evolution	[294]
	2018	Mouse model	Myeloid-specific PAD4 deletion reduced NETs release and atherosclerosis burden	Strengthens genetic evidence for NETs as drivers of disease	[295]
	2018	Mouse model	NETs promoted thrombotic complications in acute plaque erosion but not chronic atherogenesis	Highlights disease-stage-specific roles of NETs	[296]
	2019	Human autopsy (*n* = 12)	NETs dominated in early thrombosis, and macrophage traps in late thrombosis	Demonstrates temporal shift in immune trap dominance during thrombogenesis	[297]
Autoimmune diseases	2009	Human neutrophils + kidney biopsies (SVV patients)	ANCA-stimulated neutrophils released NETs containing PR3 and MPO. NETs are prominent in biopsies with neutrophil infiltration	NETs act as antigen reservoirs fueling autoantibody production	[31]
	2010	Sera from SLE/RA patients vs controls	DNase I inhibitors and anti-NETs antibodies in SLE sera blocked NETs clearance	Defective NETs degradation contributes to autoantigen persistence	[298]
	2011	SLE patient neutrophils	Type I IFN-primed neutrophils underwent NETosis upon antibody stimulation, activating pDCs and IFN-α production	Links NETs to amplification of the IFN-driven autoimmune loop	[299]
	2012	Mouse model	NET-loaded myeloid DCs promoted ANCA autoimmunity	Shows cross-talk between NETs and adaptive autoimmunity	[300]
	2012	NOX2-deficient lupus-prone mice	NETs do not contribute to lupus in the model	Suggests disease-specific dependence on NETs	[130]
	2012	PAD4-deficient arthritis mice	PAD4 deficiency did not affect arthritis severity	Indicates NETs' dependence may vary across autoimmune diseases	[301]
	2014	RA patient neutrophils	Increased spontaneous NETs release; diagnostic potential of NETs markers in RA	NETs may serve as biomarkers for inflammatory arthritis	[302]
	2015	Human endothelial cells (in vitro)	NET-associated MMP-9 activated MMP-2, driving endothelial dysfunction; inhibition restored vascular function	NETs' proteases directly contribute to vascular injury	[303]
	2015	SLE mouse model	PAD inhibition reduced NETs, protecting against lupus-related kidney/skin damage	Validates PAD inhibition as a therapeutic strategy in SLE	[246]
	2015	RA synovial fluid	Elevated extracellular DNA correlated with neutrophils and PAD activity; PAD2/4 was found in NETs	Links NETs with joint inflammation severity	[304]
	2015	Arthritis mouse model	PAD4 gene deletion reduced arthritis severity	Strengthens the causal role of PAD4-mediated NETs	[305]
	2016	MPO-ANCA mouse model	PAD inhibition suppressed NETs and autoantibody formation	Demonstrates PAD inhibitors as immunomodulators	[139]
	2017	AVV patient neutrophils + kidney biopsies	Enhanced NETs with LAMP-2 content; anti-LAMP-2 antibodies further promoted NETosis	Suggests autoantibodies amplify NET-driven damage	[306]
	2017	JIA synovial neutrophils	DEK enriched in NETs; DEK-aptamers reduced inflammation in vivo	Reveals novel therapeutic target beyond PAD inhibition	[307]
	2017	RA patient plasma	NETs elevated in RA; inhibition reduced endothelial dysfunction and immune activation	Reinforce the vascular-immune interface role of NETs	[308]
	2018	RA patient’s sera	Elevated MPO-DNA complexes correlated with neutrophil counts and autoantibodies	NETs markers may stratify disease activity	[140]
	2018	SLE neutrophils	RIPK1-deficient neutrophils are more prone to NETosis; RIPK1 inhibition reduced NETs	Identifies RIPK1 as a regulator of NETosis	[131]
Sepsis	2004	In vitro	Neutrophils released NETs that bound bacteria, degraded virulence factors, and killed pathogens	Demonstrated antimicrobial function of NETs in early sepsis	[309]
	2012	Mouse model	NETs enhanced bacterial trapping; blocking NETs worsened dissemination	Suggests a protective role in early infection control	[310]
	2012	Mouse model	DNase treatment impaired early immunity, worsening pathology	Indicates that premature NETs degradation is harmful	[311]
	2014	Septic mice	NETs enriched in microvasculature (cecum, liver, lung)	Supports role in microcirculatory dysfunction	[312]
	2016	Mouse model	DNase + antibodies attenuated sepsis-induced organ injury, improving survival	Combination therapy may balance NETs clearance vs antimicrobial defense	[313]
	2017	Mouse model	NETs promoted intravascular coagulation; inhibition improved perfusion and reduced damage	Links NETs directly to coagulopathy in sepsis	[314]
	2017	Mouse model	PAD4 inhibition (CI-amidine) improved survival post-sepsis induction	Supports therapeutic targeting of PAD4	[315]
	2017	Human neutrophils + plasma	Septic patients had higher NETs release; NETs promoted hypercoagulability	Confirms clinical relevance in sepsis-induced coagulopathy	[316]
	2018	Mouse + human ARDS patients	NETs are abundant in bacterial pneumonia/ARDS; plasma NETs correlated with severity/mortality	NETs may serve as prognostic biomarkers	[317]
	2018	Mouse model	NETs induced macrophage pyroptosis, amplifying sepsis inflammation	Reveals a new NET-macrophage death pathway link	[318]
Cancer metastasis	2013	Mouse model	NETs captured circulating tumor cells, facilitating metastasis	Identifies NETs as a physical scaffold for metastasis	[319]
	2016	Post-surgical stress mouse model	NETs promoted liver metastasis after surgery	Suggests perioperative NETs inhibition could reduce metastasis	[320]
	2018	Mouse model (dormant cancer)	Inflammation-induced NETs reawakened dormant tumor cells, driving metastasis	Demonstrates role in tumor dormancy escape	[40]
	2018	Gastric cancer metastasis mouse model	Salvianolic acid B and DHT I inhibited NETs, reducing metastatic nodules	NETs inhibition shows therapeutic promise in cancer	[321]
	2021	Clinical + preclinical studies	NETs promoted tumor cell migration and enhanced metastatic potential	Confirms the clinical significance of NETs in cancer progression	[322]

*Abbreviations: NETs* neutrophil extracellular traps, *ANCA* anti-neutrophil cytoplasmic antibodies, *PR3* proteinase 3, *MPO* myeloperoxidase, *pDC* plasmacytoid dendritic cell, *PAD4* peptidyl arginine deiminase 4, *SLE* systemic lupus erythematosus, *STEMI* ST-elevation myocardial infarction, *MI* myocardial infarction

Ongoing clinical trials are also evaluating NET-targeted strategies in respiratory conditions, such as chronic obstructive pulmonary disease (COPD), cystic fibrosis, and COVID-19, as well as in cardiovascular diseases, including atherosclerosis and thrombosis. In crystal-induced disorders such as gout and silicosis, interventions aimed at blocking NETs release are being tested to mitigate inflammation and tissue damage. Rheumatoid arthritis studies are investigating NETs both as biomarkers and as therapeutic targets to correlate NET-forming neutrophil subsets to disease severity. Collectively, these studies highlight the broad translational potential of NET-targeted interventions. By integrating diagnostic and therapeutic strategies, NETs modulation may advance precision medicine and improve outcomes across multiple disease states. Table 4 lists ongoing and completed clinical trials evaluating therapeutic strategies that directly or indirectly target NETs formation or degradation across a spectrum of human diseases.

**Table 4:** Clinical trials and translational studies targeting NETs or NET-related pathways

Diseases/Conditions	Drug/Intervention	Clinical trials ID	Study focus/Key findings	Clinical implications	Status
ARDS	Sivelestat (neutrophil elastase inhibitor)	NCT06387823	Evaluating the combined use of sivelestat sodium with dexamethasone in ARDS patients	Explores combinatorial anti-inflammatory therapy to reduce the neutrophil/NETs burden	Recruiting
		NCT04973670	Assessing the protective effect of sivelestat in ARDS associated with sepsis	Sepsis-associated ARDS in a NETs-driven pathology; NE inhibition may improve survival	Recruiting
Acute aortic dissection	Sivelestat	NCT05874700	Pilot study testing whether sivelestat reduces ventilation time in patients with aortic dissection	Potential for organ-protection during cardiovascular emergencies	Not yet recruiting
Cardiac surgery complications	Sivelestat	NCT06195267	Evaluating effects of sivelestat on postoperative pulmonary and multi-organ dysfunction	Could broaden clinical applications beyond ARDS into surgical medicine	Recruiting
Myelodysplastic syndrome	SX-682 (CXCR1/2 inhibitor)	NCT04245397	Evaluating efficacy/safety of SX-682 in MDS patients	CXCR1//2 blockade prevents neutrophil trafficking, indirectly limiting NETosis	Recruiting
Multiple myeloma	SX-682 + standard of care	NCT06622005	Combination trial with Carilzomin, Daratumumab-Hyaluronidase, and Dexamethasone	Tests the synergy between the NET-targeting existing immunotherapy	Recruiting
Metastatic melanoma	SX-682 ± pembrolizumab	NCT03161431	Safety profile analysis of SX-682 alone or combined with a checkpoint inhibitor	Combines NETs inhibition with immunotherapy to enhance T cell response	Recruiting
Pancreatic cancer	SX-682 + tislelizumab	NCT05604560	Pre-surgical use in resectable pancreatic cancer	Potential to improve surgical outcomes and reduce metastasis by lowering NETs activity	Recruiting
Pancreatic ductal adenocarcinoma	SX-682 (maintenance)	NCT04477343	Maintenance therapy trial in metastatic PDAC patients	Evaluates NETs blockade in aggressive cancer settings	Recruiting
NSCLC (stage III/IV)	SX-682 vs pembrolizumab	NCT05570825	Ongoing comparative study	Investigates whether NETs blockade enhances response to immunotherapy	Recruiting
Prostate cancer	SX-682 + enzalutamide	NCT06228053	Testing efficacy in abiraterone-resistant metastatic prostate cancer	Combines androgen receptor inhibition with NETs blockade	Recruiting
Metastatic castration-resistant prostate cancer	AZD5069 + enzalutamide	NCT03177187	Combination safety trial; terminated early	Demonstrates the challenges of combining NETs inhibition with hormonal therapy	Terminated
Ischemic stroke	Pulmozyme (Dornase alfa, rhDNase)	NCT05203224, NCT04785066	Testing adjuvant DNase to enhance reperfusion and arterial recanalization	Novel strategy to degrade prothrombotic NETs in stroke	Recruiting
Respiratory distress syndrome/Trauma	Pulmozyme	NCT03368092	Inhaled DNase reduced respiratory failure in trauma patients	Expands the use of NETs degradation to trauma-related lung injury	Recruiting
COVID-19 respiratory failure	Pulmozyme	NCT04445285	Testing DNase therapy to reduce mortality in COVID-19 ARDS	Direct translation of NETs biology to pandemic-driven ARDS	Recruiting
Pleural empyema	Pulmozyme	NCT04095676	Compared VATS vs drainage with DNase therapy	Evaluates the role of NETs clearance in pleural infections	Recruiting
Head and neck cancer	Pulmozyme + radiotherapy	NCT00536952	Assessing the efficacy of DNase during chemoradiotherapy	May mitigate NET-mediated tissue damage in cancer therapy	Recruiting
Leukemia (AML/ALL)	Oshadi D + Oshadi R	NCT02462265	Trial evaluating Oshadi formulations in chemotherapy regimens	Highlights growing interest in indirect NET-targeting via novel agents	Suspended

*Abbreviations: ARDS* acute respiratory distress syndrome, *NE* neutrophil elastase, *CXCR* C-X-C chemokine receptor, *COPD* chronic obstructive pulmonary disease, *DNase* deoxyribonuclease I, *rhDNase* recombinant human DNase, *NETs* neutrophil extracellular traps

While the therapeutic targeting of NETs holds considerable promise, several translational challenges remain. The dual role of NETs in both host defense and disease pathogenesis requires careful modulation to avoid impairing essential immune functions. Furthermore, heterogeneity in NETs composition and activity across different pathological contexts complicates the development of broadly effective interventions. Disease-specific variations in NET-inducing stimuli and clearance mechanisms necessitate tailored therapeutic strategies. As the field advances, a key priority will be to delineate the context-dependent effects of NETs and identify reliable biomarkers to guide patient stratification. Achieving this balance will be essential for optimizing NET-directed therapies and ensuring safety and efficacy in clinical settings.

## Conclusion and future perspectives

NETs have emerged as pivotal mediators at the interface of antimicrobial defense, immune regulation, and tissue remodeling. While they are indispensable for pathogen neutralization and the orchestration of early immune responses, excessive or dysregulated NETs formation drives chronic inflammation, vascular injury, autoimmunity, and cancer progression. Preserving the delicate balance between NETs generation and clearance is therefore fundamental to maintaining immune homeostasis and preventing disease.

Over the past two decades, substantial progress has been made in delineating the molecular machinery of NETosis, including NOX-dependent and -independent pathways, PAD4-driven histone citrullination, and neutrophil elastase-mediated chromatin remodeling. Yet, critical questions remain regarding the context-specific stimuli that initiate NETs formation, the mechanisms governing their clearance, and the extent of their interactions with adaptive immunity, complement, and stromal compartments. Addressing these gaps is essential for translating basic mechanistic insight into clinically meaningful strategies.

Methodological advances, such as high-resolution imaging, ELISA-based detection, flow cytometry, and single-cell sequencing, have expanded the toolkit for studying NETs in both experimental and patient settings. However, the lack of standardized assays and the challenge of distinguishing NETs from other neutrophil-derived structures continue to hinder clinical translation. Establishing robust, validated, and reproducible detection protocols will be indispensable for integrating NETs biology into diagnostic and prognostic workflows.

From a therapeutic perspective, NETs represent a double-edged sword. Preclinical and early-phase clinical studies investigating PAD4 and elastase inhibitors, DNase-based approaches, and antibody-mediated neutralization have shown promise across cancer, autoimmunity, and thromboinflammatory conditions. However, indiscriminate inhibition risks compromising host defense, underscoring the need for strategies that selectively attenuate pathogenic NETs while preserving their physiological antimicrobial functions.

Looking forward, integrating NETs research with system biology, multi-omics, and computational modeling is likely to uncover disease-specific NETs signatures and regulatory networks. Such insights, coupled with closer collaboration between immunologists, molecular biologists, and clinicians, will accelerate the translation of NETs biology into precision medicine. Ultimately, bridging the mechanistic underpinnings with clinical application holds the potential to transform NETs from biomarkers of pathology into actionable therapeutic targets.

## Data Availability

Not applicable.

## Abbreviations

AAV: Adeno-associated virus
AKI: Acute kidney injury
ANCA: Anti-neutrophil cytoplasmic antibody
ARDS: Acute respiratory distress syndrome
AVV: ANCA-associated vasculitis
CG: Cathepsin G
CTCs: Circulating tumor cells
DEK: DNA-binding protein DEK
DHTI: 15, 16-Dihydrotanshinone I
ECs: Endothelial cells
PDG: 2-Fluoro-2-deoxy-D-glucose
GM-CSF: Granulocyte–macrophage colony-stimulating factor
IFN-α: Interferon alpha
IRA: Infract-related artery
JIA: Juvenile idiopathic arthritis
MI: Myocardial infarction
MMP-9: Matrix metalloproteinase-9
MPO: Myeloperoxidase
NE: Neutrophil elastase
NEFAs: Non-esterified fatty acids
NETs: Neutrophil extracellular traps
NOX2: NADPH oxidase 2
OSM: Oncostatin M
PAD4: Peptidyl arginine deiminase 4
PAF: Platelet-activating factor
Panx1: Pannexin 1
pDCs: Plasmacytoid dendritic cells
PMA: Phorbol 12-myristate 13-acetate
PMNs: Polymorphonuclear neutrophils
PR3: Proteinase 3
RA: Rheumatoid arthritis
RCC: Renal cell carcinoma
rhDNase: Recombinant human deoxyribonuclease
RIPK1: Receptor-interacting protein kinase 1
ROS: Reactive oxygen species
Sal B: Salvianolic acid B
SLE: Systemic lupus erythematosus
STEMI: ST-elevation myocardial infarction
SVV: Small vessel vasculitis
TAN: Tumor-associated neutrophils
TINs: Tumor-infiltrating neutrophils
TME: Tumor microenvironment
